# Cancer Cells Enter an Adaptive Persistence to Survive Radiotherapy and Repopulate Tumor

**DOI:** 10.1002/advs.202204177

**Published:** 2023-01-19

**Authors:** Yucui Zhao, Tingting Lu, Yanwei Song, Yanqin Wen, Zheng Deng, Jiahui Fan, Minghui Zhao, Ruyi Zhao, Yuntao Luo, Jianzhu xie, Binjie Hu, Haoran Sun, Yiwei Wang, Sijia He, Yanping Gong, Jin Cheng, Xinjian Liu, Liang Yu, Jikun Li, Chuanyuan Li, Yongyong Shi, Qian Huang

**Affiliations:** ^1^ Shanghai Key Laboratory for Pancreatic Diseases and Cancer Center Shanghai General Hospital Shanghai Jiao Tong University School of Medicine Shanghai 201620 China; ^2^ Bio‐X Institutes Key Laboratory for the Genetics of Developmental and Neuropsychiatric Disorders (Ministry of Education) Shanghai Jiao Tong University Shanghai 200030 China; ^3^ Zhejiang Provincial Key Laboratory of Pancreatic Disease The First Affiliated Hospital Zhejiang University School of Medicine Hangzhou 310009 China; ^4^ Department of Biochemistry School of Medicine Sun Yat‐sen University Shenzhen 518107 China; ^5^ Department of General Surgery Shanghai General Hospital Shanghai Jiao Tong University School of Medicine Shanghai 201620 China; ^6^ Department of Dermatology Duke University Medical Center Box 3135 Durham NC 27710 USA; ^7^ Biomedical Sciences Institute of Qingdao University (Qingdao Branch of SJTU Bio‐X Institutes) Qingdao University Qingdao 266003 China

**Keywords:** poly‐aneuploid giant cancer cells, radiation tolerant persister, radio‐resistance, tumor repopulation

## Abstract

Repopulation of residual tumor cells impedes curative radiotherapy, yet the mechanism is not fully understood. It is recently appreciated that cancer cells adopt a transient persistence to survive the stress of chemo‐ or targeted therapy and facilitate eventual relapse. Here, it is shown that cancer cells likewise enter a “radiation‐tolerant persister” (RTP) state to evade radiation pressure in vitro and in vivo. RTP cells are characterized by enlarged cell size with complex karyotype, activated type I interferon pathway and two gene patterns represented by CST3 and SNCG. RTP cells have the potential to regenerate progenies via viral budding‐like division, and type I interferon‐mediated antiviral signaling impaired progeny production. Depleting CST3 or SNCG does not attenuate the formation of RTP cells, but can suppress RTP cells budding with impaired tumor repopulation. Interestingly, progeny cells produced by RTP cells actively lose their aberrant chromosomal fragments and gradually recover back to a chromosomal constitution similar to their unirradiated parental cells. Collectively, this study reveals a novel mechanism of tumor repopulation, i.e., cancer cell populations employ a reversible radiation‐persistence by poly‐ and de‐polyploidization to survive radiotherapy and repopulate the tumor, providing a new therapeutic concept to improve outcome of patients receiving radiotherapy.

## Graphical abstract

Cancer cells enter a radiation‐tolerant persister (RTP) state by polyploidization to survive radiation. RTP cells then de‐polyploidize and generate parent‐like progenies via viral budding‐like division resuming tumor repopulation. 10× and switching mechanism at 5′ end of the RNA transcript (SMART) single cell RNA‐seq unveil the essential role of type I interferon and two gene panels in budding phenotype, pointing to novel therapeutic strategies in radiotherapy.

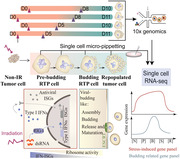

## 1 Introduction

Radiotherapy is a vital modality of cancer treatment, and approximately half of cancer patients accept radiotherapy at different period during treatment.^[^ ^1^ ^]^ However, development of radio‐resistance has emerged as a major cause of therapy failure as well as local or distant relapses.^[^ ^2^ ^]^ Tumor repopulation is considered particular important in clinical practice because the treatment dose is always given in fractions no matter in conventional, hyper‐ or super‐ segmentation manner. During irradiation intervals, surviving tumor cells potentially resume accelerated proliferation and eventually repopulate the tumor.^[^ ^3^ ^]^ Multiple mechanisms of repopulation have been elucidated, such as hypoxia, cancer stem cells (CSCs), etc. We have reported that radiation‐induced apoptotic cells can stimulate proliferation of survived tumor cell via caspase‐3/ca^2+^‐independent phospholipase A_2_/arachidonic acid/prostaglandin E_2_ paracrine pathway.^[^ ^4^ ^]^ However, much remains to be figured out about the subset of tumor cells capable of repopulating and the key molecular mechanisms involved.

It has been increasingly appreciated that cancer cells can enter a “drug‐tolerant persister” (DTP) state to evade death from chemo‐ or targeted therapy.^[^ ^5^ ^]^ Analogous to the concept of “bacterial persister cells” in response to antibiotics, DTP cells survived anticancer agents by slowing down cycling, later initiated growth and restored drug‐sensitive state upon drugs withdrawal.^[^ ^5^, ^6^ ^]^ Characterized with cell metabolism flexibility, microenvironment adaptation and phenotypic plasticity, chemo‐ or targeted therapy induced DPT has ever been recognized by non‐genetic mechanisms and may act as reservoir for long‐term genetic resistance.^[^ ^7^ ^]^ Studies of Russo et al. reported that targeted therapy induced downregulation of high‐fidelity DNA damage repair and upregulation of error‐prone polymerases in human colorectal cancer (CRC), indicating the parallel between DTP cells and bacteria persister cells in the ability to evade therapeutic pressure by enhancing adaptive mutagenesis.^[^ ^5b^ ^]^ In stress of ionizing radiation that induces cell‐killing by breaking DNA, we and others also detected residual radiation‐tolerant cancer cells, which were capable of recovering proliferation upon cessation of radiation and driving tumor repopulation. Interestingly, we found this radiation‐tolerance was accompanied with features of poly‐aneuploid cancer cells (PACCs), i.e., enlarged cellular morphology and aberrant nuclei as well as extremely complex karyotypes. The tolerant state was relinquished when majority of cancer cells returned normal morphology and karyotype. Furthermore, the radiation‐tolerant PACCs will form again when facing next round of irradiation. Based on this fact, we termed this transient persistence of radiation‐induced giant cancer cells as “radiation‐tolerant persister” (RTP) by analogy to DTP state. We also wondered whether and how RTP cells contribute to tumor repopulation.

Because of their prominent morphology and enrichment during anticancer treatment or genetic stress, PACCs were early noticed by pathologists but have long been overlooked since they were recognized as senescent or mitotic catastrophic doomed to death. Recently, PACCs have been proposed as the “evil roots of cancer” with mounting evidences showing their potent ability of survival, stemness and resistance to oncotherapy.^[^ ^8^ ^]^ PACCs have been proposed to originate from endoreplication, mitotic failure or abortive cytokinesis, especially in the cells with dysfunctional p53 (TP53)^[^ ^9^ ^]^ and their frequency is correlated with advanced tumor staging and worse prognosis.^[^ ^8^, ^10^ ^]^ Traditional molecular methods used to analyze PACCs only obtained average information, precluding the identification of those PACCs with repopulating potentials. The advent of single‐cell sequencing technologies and bioinformatics methods has enabled dissection of cell fate dynamics at the individual‐cell resolution.

Here, using CRC and other cancer cell lines, cancer cell line‐derived xenografts and patient‐derived xenografts (PDX) models, we revealed that RTP cells could be induced by radiotherapy, and they could produce progenies via a viral budding‐like division. Indeed, these progeny cells gradually recovered back to a chromosomal constitution similar to their unirradiated parental cells, participating in tumor repopulation. Furthermore, we performed single cell SMART‐sequencing (switching mechanism at 5′ end of the RNA transcript), 10× platform and functional studies to dissect molecular characteristics of RTP states and identify key signaling and gene profiling involved in budding of RTP cells.

## 2 Results

### 2.1 Evidence for Presence of Radiation‐Tolerant Persister State In Vitro and In Vivo

**Figure 1:**
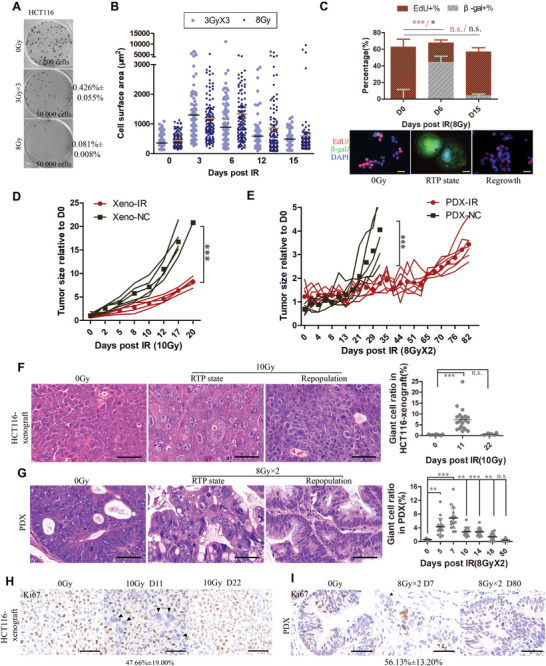
Detection of radiation‐tolerant persister state in vitro and in vivo. A) Untreated (200 cells), 3 Gy × 3 and 8Gy‐irradiated (50000 cells) HCT116 were seeded in 6‐well plate and shown are representative images of colonies formed. B) Dot plot showing change of cell surface area with time post irradiation with indicated doses. Each point represents one cell and at least 110 cells were counted. C) Histogram showing the alteration of proliferation and senescence activity by co‐staining of EdU (red) and *β*‐gal (green) at indicated time post 8 Gy. Scale bar, 25 mm. D) Mean tumor size relative to baseline of day 0 for xenograft of HCT116 cells treated with 10 Gy and E) for an 8 Gy × 2‐irradiated xenograft model derived from a moderately differentiated rectal adenocarcinoma patient. *n* = 5 for each group; Two‐way ANOVA. F,G) H&E immunohistochemistry staining showing the formation of giant cancer cells during radiation‐tolerant persister (RTP) state in 10Gy‐treated HCT116 xenograft (F, middle picture) and 8 Gy × 2‐irradiated PDX model (G, middle picture) comparing to untreated and repopulated tumor. Scale bar, 25 mm. Dot plots depict ratio of giant cells (mean ± SEM); one‐way ANOVA. H,I) Immunohistochemistry analysis (staining of Ki67) of corresponding samples in panels (F,G). Ki67 negative (black triangle) giant cancer cells are indicated. Scale bar, 50 mm. *p < 0.05,**p < 0.01, ***p < 0.001.

We performed clonogenic cell survival assay to evaluate the features of tumor repopulating cells following irradiation. Using either a fractionated (3 Gy per day for 3 days) or a single dose (8 Gy) irradiation regimen that killed the vast majority of HCT116 cells, a small fraction of cancer cells could survive and grow into colonies (**Figure** **1**A). Unlike chemo‐ or targeted therapy, the most common morphological change post irradiation is cellular enlargement which almost involves all cells although majority of enlarged cells will eventually die. Average sizes of both 3 Gy × 3 and 8Gy‐irradiated cells showed a trend of first increasing (>threefold change) and then decreasing to unirradiated baseline (Figure 1B). Relative to 3 Gy × 3 regimen, cells irradiated with 8 Gy formed fewer colonies with prolonged duration of cellular enlargement. For these residual giant cancer cells, their senescence‐like feature characterized by positive *β*‐galactosidase activity and mostly negative EdU staining reverted to proliferative normal‐sized state when the colony was re‐formed (Figure 1C). We refer to the population of giant cancer cells with reversible dormancy as “radiation‐tolerant persister cells” (RTP cells).

In xenografts of HCT116 cells irradiated with a single 10 Gy and a rectal adenocarcinoma patient‐derived xenografts (PDX) treated with fractionated regimen of two times 8 Gy, we noticed similar profiles of tumor response to irradiation in both xenografts, where tumor eventually progressed after an initial period of growth delay versus control (Figure 1D,E). Giant RTP cells, characterized by enlarged cell sizes with swelling mono‐ or multi‐nuclei, were induced initially and decreased with tumor relapse (Figure 1F,G). Results of Ki67 staining in irradiated HCT116‐xenograft and PDX further unveiled the reversibility from non‐proliferative giant RTP cells to proliferative repopulated cells (Figure 1H,I).

### 2.2 RTP Cells were Phenotypic Plastic and Tumorigenic Independent of Stemness

**Figure 2:**
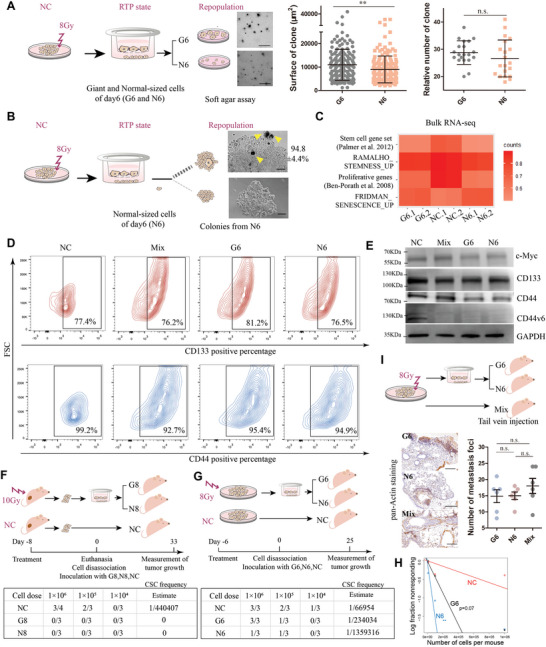
RTP cells were phenotypic plastic and tumorigenic independent of stemness. A,B) Scheme illustrating that giant (G6) and normal‐sized cells (N6) were isolated from 8Gy‐irradiated HCT116 on day 6 by mesh filtering. A) Potentials of soft agar colony formation were compared between G6 and N6. (Left panel) shown are typical images of colonies formed. Scale bars, 250 µm. Number of colonies and surface of colonies were analyzed in middle and right panel, respectively. Mean ± SEM; Student's *t* test. B) Giant cell (yellow triangle) could be found in colonies formed from single N6 cells. Scale bars, 100 µm. C) Heat map of gene signatures expressed in 8Gy‐induced G6 and N6 versus unirradiated (NC) cell samples. N = 2 for each group. D) CD133 and CD44 expression in unsorted (Mix) cells on day 6 following 8 Gy, G6, N6, and NC cells detected by cytometry analysis. E) Expression of cancer stem cell markers in Mix, G6, N6, and NC cells detected by western blot. F) Diagram of the in vivo limited dilution assays (LDA). G,H) Diagram of the simplified LDA assay. Comparison of cancer stem cell (CSC) frequency among G6, N6, and NC cells. *t*‐test of G6 versus NC and N6 versus NC are indicated. I) Representative immunohistochemistry pictures displaying the presence of metastasis in the lung tissue formed by G6, N6, and Mix. Scale bar, 200 mm. Right panels: numbers of lung metastasis foci (>50 pan Actin‐positive cells) were counted. Mean ± SEM; One‐way ANOVA. *p < 0.05, **p < 0.01, ***p < 0.001; n.s., not significant.

We noticed that giant cancer cells were enriched in RTP state and could also be detected in the repopulating colonies (Figure 1B,C; Figure S1A, Supporting Information). Similar phenomenon was consistently observed in other irradiated cancer cell lines (Caco‐2, A549 and MDA‐MB‐231 cells, Figure S1B–D, Supporting Information), suggesting the general relevance of giant cells with cancer recovery from irradiation. We further utilized nylon meshes to sort out 8Gy‐induced giant (G6) and normal‐sized (N6) cancer cells on day 6 and seeded them simultaneously in soft agar. Sizes of colonies were larger in irradiated G6 than in irradiated N6, despite that no difference was shown in the number of colonies (**Figure** **2**A). Notably, during formation of colonies derived from single N6 cells, almost all colonies (94.8%±4.4%) contained giant cancer cells (Figure 2B). The colonies consisted of pure small cells accounted ≈4% and were much smaller than colonies containing giant cells (Figure 2B). These results suggested the delayed enlargement of N6 and the clonogenic potential of RTP cells in vitro.

CSCs, characterized by their intrinsic resistance to cytotoxic therapy and unlimited self‐renewal potential, are recognized as the important constituent of tumor repopulating cells.^[^ ^11^ ^]^ We thus wondered whether CSCs are enriched in RTP population. Results from RNA sequencing of untreated HCT116, 8Gy‐treated G6 and N6 cells confirmed the persistence^[^ ^12^ ^]^ of G6 but argue against selective enrichment for embryonic and adult stem cell‐like stemness^[^ ^13^ ^]^ in RTP cells (Figure 2C). Via flow cytometry (Figure 2D) and western blot analysis (Figure 2E), we did not detect remarkable enrichment of typical CSC markers (including CD133, CD44 (CD44v6) and c‐Myc) in G6 cells compared with N6 and unirradiated cells.

In view of the clonogenic ability of RTP cells in vitro, we next performed limited dilution assays (LDAs) to evaluate the repopulation potential of giant RTP cells in vivo.^[^ ^11^, ^14^ ^]^RTP cells (G8), normal‐sized cells (N8) dissected from 10Gy‐irradiated primary tumor on day 8 or untreated primary cells (NC) were re‐inoculated in nude mice as a limited dilution series (Figure 2F). The cells from unirradiated primary tumor showed high incidence and rapid growth, whereas we did not observe tumor formation of G8 or N8 cells even in highest dose from primary tumor‐derived, irradiated cells (Figure 2F; Figure S1E, Supporting Information). In the simplified LDA assay, where inoculated cells were derived from 8Gy‐irradiated cultured cells, we observed the in vivo repopulating potential of RTP cells, but their tumor incidence was seen much later than NC cells (Figure S1F, Supporting Information). In addition, the self‐renewal frequency of G6 cells was 71% lower than untreated cells (Figure 2G‐H). We also carried out intravenous injection assay in mice with identical number of 8Gy‐irradiated unsorted (mixed), G6 and N6 cells (Figure 2I). Comparable numbers of micro‐metastasis foci in lung tissue were found in three groups, confirming the tumorigenic potential of irradiation‐induced RTP cells in vivo. Altogether, the data suggest that radiation‐induced RTP cells serve as bad seeds to initiate tumor repopulation and appear not related to stemness profiles.

### 2.3 Poly‐Aneuploid RTP Cells Contributed to Tumor Repopulation in A Viral‐Budding Mimicry Manner

**Figure 3:**
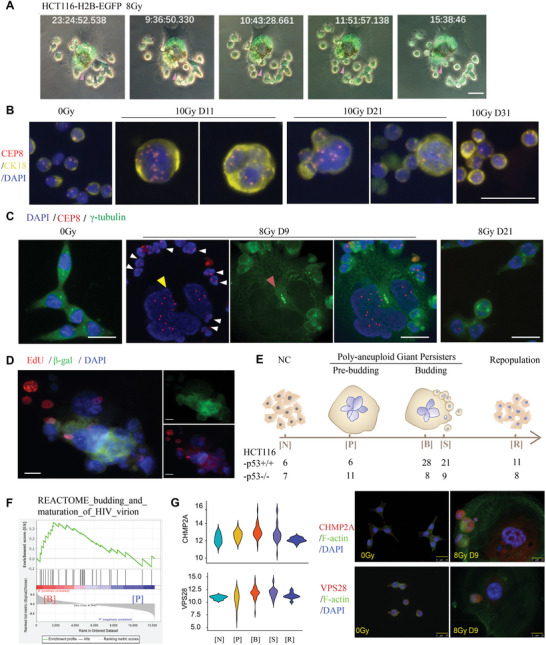
Poly‐aneuploid RTP cells produced proliferating progenies via a viral budding‐like cell division. A) Time‐lapse sequences illustrating budding of an HCT116‐RTP cell expressing H2B‐EGFP following 8 Gy. A daughter cell (pink triangle) is being budded off. Scale bars, 25 µm. B) Representative iFISH images of HCT116 cultivated in suspension illustrating the alteration of ploidy number at indicated time points following 10 Gy. Ploidy number was visualized by CEP8 probe (human chromosome 8), DNA and cytokeratin was stained by DAPI (blue) and CK18 (golden). Scale bars, 25 µm. C) Copy numbers of chromosome 8 (CEP8, red) and centrosome (*γ*‐tubulin, green) were detected by iFISH in unirradiated and 8Gy‐treated HCT116. (Middle): A RTP cell (yellow triangle) in process of budding is surrounded by diploid progeny cells (white triangle), which is centrosome (red triangle) independent. (Right): A repopulated tumor cell is undergoing mitosis. D) Immunofluorescent staining of budding RTP cells by EdU (red), DAPI (blue) and senescence‐associated *β*‐gal (green). E) Schematic of the naming principle and cell count of cells samples collected for SMART RNA‐seq. Single cells from untreated HCT116 (both p53+/+ and p53−/−) cells were defined as [N], while cells derived from pre‐budding RTP cells, budding RTP cells, budded and repopulated small cells were, respectively, defined as [P], [B], [S], and [R]. F) Gene set enrichment analysis (GSEA) showing positive enrichment for budding and maturation of human immunodeficiency virus (HIV) virion in budding RTP cells relative to pre‐budding RTP cells. G) Expression of ESCRT genes CHMP2A and VPS28 in violin plots and in immunofluorescent staining. Scale bar, 25 mm. iFISH: immunofluorescence in situ hybridization; ESCRT: endosomal sorting complex required for transport.

In next tracking H_2_B‐EGFP expressing giant cells of 8Gy‐treated HCT116, we observed that some RTP cells sprouted and budded off progeny cells resembling viral budding (**Figure** **3**A). 3D culture of HCT116 clearly demonstrated formation of poly‐aneuploid RTP cells with increased DNA contents. Subsequent budding of RTP cells generated diploid progenies, which eventually resumed to size of unirradiated cells (Figure 3B; Figure S1G, Supporting Information). Co‐labeling CEP8 with filamentous actin (F‐actin) or with CEP12 confirmed the karyotype restoration of budded progenies to their unirradiated parental cells (Figure S1H,I, Supporting Information). Irradiation‐induced poly‐aneuploid RTP cells of Caco‐2, A549 and MDA‐MB‐231 cells also underwent depolyploidization (i.e., ploidy reduction) by budding off progenies with fairly normal CEP8 numbers (Figure S1J, Supporting Information). Compared with unirradiated and repopulated diploid cells, where centrosome‐functional mitosis could be observed, budding of 8Gy‐induced RTP cells was mitosis‐distinct with multiple centrosomes clustered in the perinuclear cytoplasm (Figure 3C). Furthermore, we detected unexpectedly co‐expressions of the senescence marker with the proliferating marker in budding RTP cells (Figure 3D).

To gain insight into the karyotypic reversibility of RTP cells, we applied SMART RNA‐sequencing to obtain the longitudinal transcriptional profiles of their different stages, including unirradiated cells [N], pre‐budding RTP cells [P], budding RTP cells [B] and newly budded progeny cells [S], as well as repopulated cells [R] (Figure 3E). A total of 115 single‐cell transcriptomes (69 from HCT116 p53+/+ and 46 from HCT116 p53−/− cells) were obtained, with a mean of 85.5% reads mapping to the reference human genome. Besides the morphological similarity between RTP cells budding and viral budding, enriched pathways from pre‐budding to budding RTP cells were remarkably similar to those involved in the assembly and budding of human immunodeficiency virus (HIV) virions (Figure 3F; Figure S2A, Supporting Information). Thereinto, proteins of endosomal sorting complex required for transport (ESCRT) in host cells are hijacked by HIV to package and release their virions; similarly, ESCRT proteins were highly expressed in budding RTP cells, especially enriched in membrane scission sites (Figure 3G; Figure S2B, Supporting Information). Consistent with upregulated pathways in HIV re‐activation relative to latently infection,^[^ ^15^ ^]^ budding RTP cells also showed upregulation in expression of ribosomal proteins relative to pre‐budding RTP cells, corresponding to active metabolism of RNA and protein (Figure S2C, Supporting Information).

### 2.4 Type‐I Interferon Mediated Anti‐viral Signaling Suppressed Budding of RTP Cells

**Figure 4:**
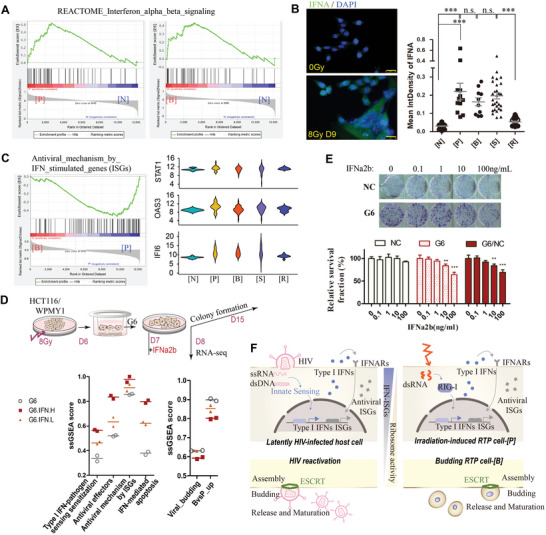
Type‐I interferon mediated anti‐viral signaling suppressed budding of RTP cells. A) GSEA showing positive enrichment for type‐I interferon signaling in pre‐budding (left panel) and budding RTP cells (right panel) relative to unirradiated cells. B) Protein expression of IFNA (green) in unirradiated and 8Gy‐treated HCT116 RTP cells. Mean IFNA fluorescence intensity of the indicated cells were shown. Scale bar, 25 mm. C) Budding RTP cells show reduced antiviral mechanism by interferon stimulated genes (ISGs) versus pre‐budding RTP cells. Violin plots showing dynamic expression of ISGs: STAT1, OAS3 and IFI6 (right panel). D) Timeline of treatment schedule: administration of low (0.1 ng mL^−1^, IFN‐L) or high (10 ng mL^−1^, IFN‐H) concentration of IFNa2b to HCT116‐RTP cells (sorted from day 6 post 8 Gy, G6) for 1 day to be collected for bulk RNA‐seq and for 7 days in colony formation assay. Single sample GSEA score of G6, G6 treated with IFN‐L or IFN‐H in indicated gene signatures (lower panel). E) Effect of IFNa2b on survival fraction of G6 and unirradiated HCT116. Mean ± SEM; One‐way ANOVA. F) Scheme showing parallels between HIV re‐activation from latency and budding of RTP cells induced by radiation. Innate viral sensing initiates production of IFN‐I and its antiviral effector ISGs, which is similar to activation of IFN‐ISGs signaling in RTP cells. Suppression of IFN‐ISGs and increase of ribosomal activity are observed both in budding of virus and RTP cells. ***p < 0.001; n.s., not significant. [N], unirradiated cells; [P], pre‐budding RTP cells; [B], budding RTP cells.

We further compared transcriptional profiles among [P], [B], and [N] cells to identify potential molecular networks accounting for the transition from mitosis to budding‐like division. Relative to unirradiated cells, pre‐budding and budding RTP cells were both characterized by marked elevation of type‐I interferon (IFN‐I) signaling (**Figure** **4**A,B; Figure S2D, Supporting Information). Indeed, signaling of retinoic acid‐inducible gene I (RIG‐I, encoded by gene DDX58), which senses viral and host‐derived dsRNA and induces transcription of IFN‐I,^[^ ^16^ ^]^ was enriched in [P] and [B] (Figure S2E, Supporting Information). Although antiviral module induced by interferon‐stimulated genes (ISGs) was both expressed in [P] and [B], ISGs expressions were transiently downregulated from pre‐budding to budding RTP cells (Figure 4C; Figure S2F,G, Supporting Information). This downregulation of antiviral activity has been reported in HIV re‐activation from its latent infective state,^[^ ^17^ ^]^ pointing to the hypothesis that RTP‐involved stress state might bear a resemblance with the host response to viral infection.

To assess the role of IFN‐I in budding of RTP cells, we treated 8Gy‐induced RTP cells with IFN*α*2b from 0.1 to 100 ng mL^−1^ (Figure 4D). Survival fraction of plated RTP cells displayed a concentration‐dependent reduction to IFNa2b versus untreated baseline, whereas IFN*α*2b did not show significant effect on survival of unirradiated HCT116 cells or stromal myofibroblast WPMY1 cells (Figure 4E; Figure S2H, Supporting Information). We next performed bulk‐RNA sequencing to compare the transcriptional profiles of RTP cells treated with 10 ng mL^−1^ of IFNa2b, with those treated at a lower concentration (0.1 ng mL^−1^) or untreatment of IFNa2b. Single sample gene set enrichment analysis (ssGSEA)^[^ ^18^ ^]^ demonstrated that exposure of IFNa2b to RTP cells triggered stronger IFN‐responsive pathways^[^ ^19^ ^]^ than untreated group, with high concentration of IFNa2b (IFN‐H) group had more significant differences (Figure 4D). Interestingly, when comparing with viral budding gene set and upregulated genes in [B] versus [P], RTP cells treated with IFN‐H had lower score, indicating that IFNa2b attenuates virus‐like budding of RTP cells by strengthening the antiviral potential.

We used several published datasets as reference, including samples of 12 tumor models pre‐ and post‐drug treatment.^[^ ^20^ ^]^ ssGSEA revealed that residual DTP cells through cytotoxic chemotherapy displayed positive enrichment in IFN‐I‐induced antiviral module and negative enrichments of genes involved in life cycle of HIV virion (Figure S2I, Supporting Information). Transcriptional profiles of chemo‐persistent tumor cells resembled our pre‐budding RTP cells, indicating generalizability of the negative feedback between antiviral signaling and viral‐mimicry budding potential in residual tumor cells after cytotoxic treatment. As summed up (Figure 4F), HIV latently infected host cells and radiation‐induced RTP cells share similar activation of IFN‐I‐mediated antiviral ISGs signaling. Transient suppression of IFN‐ISGs and enhancement of ribosome activity are involved in the virus‐like budding of RTP cells.

### 2.5 Transcriptomic Signatures of Pre‐Budding and Budding RTP Cells

**Figure 5:**
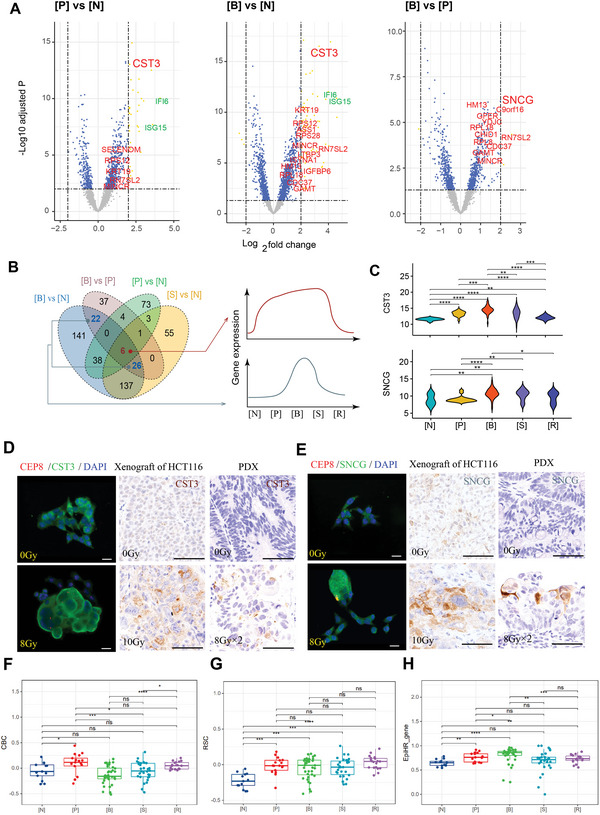
Transcriptomic signatures of pre‐budding and budding of RTP cells. A) Volcano plots displaying differentially expressed genes (DEGs, yellow dots, >fourfold and adjusted *p*<10^−2.5^) of [P] versus [N], [B] versus [N] and [B] versus [P]. Interferon signaling genes (green) and genes of interest (red) are highlighted. B) Venn diagram showing shared and differentially expressed genes (DEG, >fourfold and adjusted *p*<0.01) in four comparative groups. The 6 overlapped genes (red) and 48 genes (blue), respectively, correspond to the first and second gene expression panels. C) Expression signatures of CST3 and SNCG; Kruskal‐Wallis test. D,E) Typical expression of CST3 and SNCG in untreated and 8Gy‐induced RTP cells of HCT116 in vitro (by fluorescence staining, left panel), 10Gy‐treated xenograft of HCT116 (by immunohistochemistry, middle panel) and 8 Gy × 2‐treated PDX (by immunohistochemistry, right panel). Scale bar, 25 mm. F‐G) Using the stem cell index to map different cell stages. Kruskal–Wallis test. H) ssGSEA score of 5 cell stages in enrichment of EpiHR gene signatures. Kruskal–Wallis test. *p < 0.05, **p < 0.01, ***p < 0.001. [N], unirradiated cells; [P], pre‐budding RTP cells; [B], budding RTP cells; [S], budded progenies; [R], repopulated cell. CBC, crypt‐base columnar cells; RSC, regenerative stem cells.

To identify molecular signatures primed toward budding of RTP cells, we analyzed the differentially expressed genes (DEGs, at least fourfold change and adjusted *p* <0.01) among pre‐budding, budding RTP cells and unirradiated cells (**Figure** **5**A). Consistent with data above, genes upregulated in pre‐budding or budding RTP cells versus unirradiated cells are mainly involved in IFN‐I mediated antiviral signaling. The Venn plot further depicts two transcriptional waves through process of RTP cells budding (Figure 5B; Table S1, Supporting Information). The first gene panel is represented by CST3 and its expression is supposed to be involved in RTP cells maintenance; the second gene panel, represented by gene SNCG, also appears to be positively correlated with the budding phase (Figure 5B,C; Figure S3A, Supporting Information). Consistently, budding RTP cells with multiple copies of CEP8 have strongly up‐regulated CST3 and SNCG expression in HCT116, Caco‐2, A549 and MDA‐MB‐231 cells (Figure 5D,E; Figure S3B, Supporting Information). Irradiated xenografts of HCT116 and PDX also showed remarkably higher levels of CST3 and SNCG in RTP cells compared with untreated counterparts (Figure 5D,E). Transcriptional and protein expression of other genes were also confirmed in budding RTP cells (Figure S3C,D, Supporting Information). As for relationships among the genes of gene panel, the 6 genes of the first gene panel are not enough to enrich into any pathways, while the 48 genes of the second panel were significantly enriched into signaling of “metabolic process”, “developmental process” and “response to stimulus”, suggesting an active metabolism during budding (Figure S3E, Supporting Information).

Besides these molecular adaptations, an upregulation of embryonic diapause‐like module, displaying inactivation of MYC, increased expression of chemotherapy‐induced stress genes and diapause‐expressing genes,^[^ ^21^ ^]^ and a downregulation of cell‐cycle and proliferative genes,^[^ ^12a^ ^]^ accompanied by enrichment in senescence^[^ ^12b^ ^]^ and senescence‐associated inflammatory genes^[^ ^22^ ^]^ were observed in budding RTP cells compared to unirradiated cells (Figure S3F,G, Supporting Information). Consistent with gene‐expression signature from bulk RNA‐seq analysis, budding RTP cells did not exhibit enrichment for stemness genes^[^ ^13^, ^23^ ^]^ in single cell level (Figure S3H, Supporting Information). GSEA also suggested inactivation of embryonic stem cell signaling, Hedgehog as well as Notch4 program in budding RTP cells (Figure S3I, Supporting Information). RNA and protein expression of CSC markers (CD44 or CD44v6, CD133 and c‐myc) were significantly higher expressed in RTP state than untreated baseline of HCT116, A549 and MDA‐MB‐231 cells, as well as 10Gy‐treated HCT116‐xenograft (Figures S3J–L and S4A–F, Supporting Information). Taking into account that CD44 and CD133 have substantial expression in untreated cells that cannot all be CSCs, their elevation in RTP state is supposed not so much the feature of cancer stemness than a critical factor supporting survival of RTP cells. Negative staining of CD44v6 in unirradiated as well as RTP state of PDX further argue against stemness enrichment in RTP (Figure S3M, Supporting Information).

Interestingly, when using the stem cell index of intestinal tumor^[^ ^24^ ^]^ to map our single cell transcriptome data, we found that irradiated cells (including [P], [B], [S], and [R]) were relatively more enriched toward “regenerative stem cells” (RSCs) rather than the classical LGR5+ crypt‐base columnar cells (CBCs) (Figure 5F,G). Noted that RSCs function early in damaged intestinal epithelium,^[^ ^24^, ^25^ ^]^ distinguished from CBCs underpinning intestinal homeostasis in untreated condition, this RSC‐phenotype of budding RTP cells is more in line with their survival adaptation facing therapeutic pressure and may explain their negative enrichment in conventional stemness. Furthermore, RTP cells, especially budding RTP cells also exhibited significant EpiHR signature^[^ ^26^ ^]^ (Figure 5H), a recently established epithelial‐specific high‐risk gene set highly associated with disease relapse and poor prognosis. Collectively, our data identified RTP cells as dormant seeds with increased resistance toward irradiation and potential causing tumor relapse.

### 2.6 Gene Profiling Verified by 10× scRNA‐seq and Functional Studies

**Figure 6:**
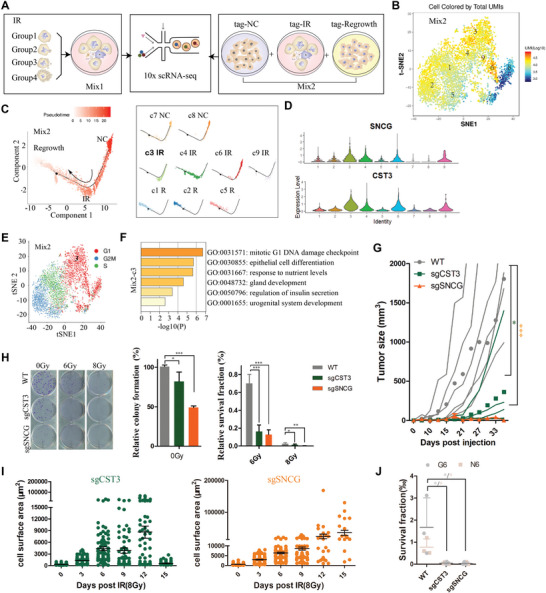
Further verification by 10 × scRNA‐seq and by functional studies. A) Single‐cell RNA‐seq pipeline. The first sample “Mix1”: consisting of distinct groups from different stages of budding RTP cells. The second sample “Mix2”: including repeated setup of “Mix1” (incubated with antibody “tag‐IR”), untreated cells (labelled as “tag‐NC”) and repopulated cells on day 30 post 8 Gy (labelled as “tag‐R”). B) t‐SNE projections of single‐cell RNA profiles in “Mix2”. Cells were colored by total unique molecular identifiers (UMI). C) Pseudo‐time reconstruction of “Mix2”. D) Significantly high expression of SNCG and CST3 in cluster 3 of “Mix2”. E) t‐SNE projections of “Mix2” single cells colored by indicated cell cycle phases. Cluster 3 was located in G1 phase. F) Gene Ontology (GO) enrichment of cluster 3 in “Mix2”. G) Tumorigenic potential in vivo among untreated HCT116 cells, its CST3 and SNCG knockout cells. *n* = 5 for each group; Two‐way ANOVA. H) Colony formation capacity of wild type HCT116 cells and its CST3 or SNCG knockout cells in indicated conditions. Mean number of colonies (± SEM, left panel) and mean survival fraction (± SEM, right panel) were shown. Student's *t*‐test (left panel); Two‐way ANOVA (right panel). I) Change of cell area post irradiation in CST3 and SNCG knockout cells. J) Colony formation potential of giant (G6) or normal‐sized cells (N6) from irradiation‐treated HCT116 cell, its CST3 and SNCG knockout cells; Student's *t*‐test. *p < 0.05, ***p < 0.001.

To mitigate potential bias due to the small number of samplings in SMART RNA‐seq, we further employed 10× Genomics scRNA‐seq to identify characteristics of RTP cells budding. Cell groups being in the period around budding of RTP cells were mixed and constructed as one sequencing library (“Mix1”). For repeating and comparing purpose, “Mix2” were performed with irradiated RTP cells (incorporated with hashtag “tag‐IR”), non‐irradiated with “tag‐NC” and repopulated cells with “tag‐R” (**Figure** **6**A; Figure S5A, Supporting Information). After quality control and removal of hashtags of ineffective‐labelled cells, we obtained 1563 individual cells in Mix1 and 4596 in Mix2 consisting of 421 unirradiated, 2976 irradiated, and 1099 repopulated cells. We first integrated Mix1 and Mix2 as “Merge1&2” and principal component analysis (PCA) showed the repeatability and reliability of our models (Figure S5B, Supporting Information).

Using t‐distributed stochastic neighbor embedding (t‐SNE) plots to project Mix2 and Mix1, we identified 9 and 13 clusters, respectively (Figure 6B; Figure S5C, Supporting Information). We next followed these clues and found cluster3 of Mix2 was the budding cluster: 1) its total genes and total unique molecular identifiers (UMI) numbers were in the top rank of clusters (Figure 6B); 2) its differential trajectory was at a transitional state (Figure 6C); 3) SNCG and CST3 were highly expressed (Figure 6D). Cluster4 of Mix1 was similarly designated as the optimal subset (Figure S5C,D, Supporting Information). We then carried out cell cycle analysis and found that these budding clusters were consistently in G1 phase, suggesting that budding‐like division occurred in a mitosis‐independent manner (Figure 6E; Figure S5E, Supporting Information). Moreover, these cells were enriched in Gene Ontology (GO) terms “developmental growth”, “cell differentiation”, “response to nutrient” and “wounding or oxygen level” (Figure 6F; Figure S5F, Supporting Information), indicating that budding RTP cells share characteristics of cellular survival under genotoxic stress.

To further validate the roles of two budding‐associated gene panels, we performed functional studies focusing on the represented genes CST3 and SNCG. CRISPR‐based knockout of CST3 or SNCG in HCT116 significantly suppressed the colony formation capacities, elevated tumor sensitivity to irradiation and inhibited the subcutaneous tumorigenicity in vivo (Figure 6G,H; Figure S5G, Supporting Information). Knockout of CST3 and SNCG in other cell lines exhibited the same reduction in colony formation ability (Figure S5H–J, Supporting Information). In tracing cellular areas after 8 Gy, we found that knockout of CST3 or SNCG could not attenuate formation of RTP cells, but suppressed generation of progeny cells, especially in irradiated sgSNCG cells (Figure 6I). We further isolated giant and normal‐sized cells in knockout cells on day 6 post 8 Gy and seeded them at equal numbers. Survival fraction of irradiated giant or normal‐sized cells both in sgCST3 and sgSNCG were significantly decreased versus irradiated wild‐type cells (Figure 6J). These results indicated that loss of function for CST3 and SNCG can suppress tumor repopulation via attenuation of budding priming.

### 2.7 Reversibility of RTP State

**Figure 7:**
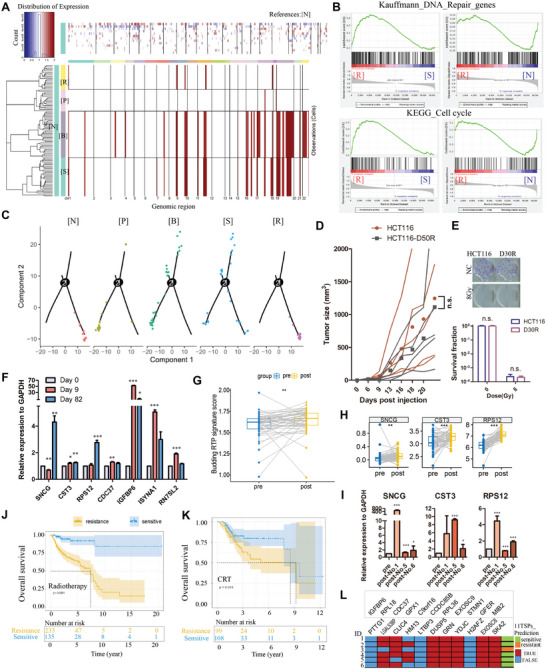
Reversibility of RTP state and clinical value of the budding gene signature. A) Clustered heat map of SMART RNA‐seq data showing chromosomal copy‐number profiles relative to untreated samples. Red, copy number gain; blue, copy number loss. B) GSEA showing positive enrichment for DNA repair genes and cell cycle genes of repopulated cells versus budded progeny cells, but negative enrichment versus untreated cells. C) Trajectory analysis for each longitudinal stage of sample groups. D) Tumorigenic potential in vivo of unirradiated or repopulated HCT116 (expanded from day 50 post 8 Gy). *n* = 5 for each group; Two‐way ANOVA. E) Representative images of colony formation in unirradiated and repopulated cells on day 30 post 8 Gy. Histogram showing the survival fractions of corresponding groups. Mean ± SEM; Two‐way ANOVA. F) RT‐PCR detection of budding RTP genes in PDX samples at 3 time points following 8 Gy × 2. Expression levels of shown genes were normalized to internal GAPDH of each samples. Mean ± SD; One‐way ANOVA. G‐H) Paired box diagrams showing the increase of budding RTP signature score and [B]_type genes expression of 52 LARC patients following chemo‐radiation (published data in GSE94104 and GSE190826). Student's *t*‐test. I) Transcriptional expression of budding RTP genes were analyzed by RT‐PCR using total RNA from 3 paired specimens of patients (pre‐ and post‐CRT of No.1, 5, 6 patient) normalized to GAPDH of each specimen. Mean ± SD; Student's *t*‐test. J,K) Kaplan‐Meier plot for cancer patients received radiotherapy J) and concurrent chemo‐radiotherapy (CRT) K) from the Cancer Genome Atlas (TCGA) database, stratified to resistant and sensitive groups according to their individual KTSP scores grounding on budding gene panel. L) 11TSPs results for 6 rectal cancer patients we collected. Each row represents one patient and each column is one of the 11 gene pairs (blue = resistant vote, red = sensitive vote). Patients who have more than 6 votes were predicted as good prognosis. [N], unirradiated cells; [P], pre‐budding RTP cells; [B], budding RTP cells; [S], budded progenies; [R], repopulated cell.

To address the question how repopulated cells ([R]) related to the newly generated progenies ([S]) and the unirradiated cells ([N]), inferCNV was firstly performed to estimate the copy number variation (CNV) of each cellular stage (**Figure** **7**A). A substantial amplification of chromosome segments occurred in budding RTP cells, and the newly budded progenies mostly inherited the CNV features of budding RTP cells, such as gain of chromosome 8–12 and 16–22. Notable was the restored karyotype of repopulating cells, whose CNV contents were closer to parental status than budded progenies. We further searched for the amplified regions remained in repopulating cells and GO enrichment analysis revealed that those multiple‐copied genes were correlated with “membrane trafficking” “response to radiation” “DNA repair” and “processing of RNA and proteins” (Figure S6A, Supporting Information), indicating that repopulated tumor cells were actively trying to repair DNA and this may be responsible for the restoration of their karyotype to those of parental cells. Furthermore, GSEA showed that molecular profiles of DNA repair and cell cycle were strongly suppressed in budded progenies [S] versus repopulated [R] cells, which were also suppressed in repopulating [R] cells versus unirradiated [N] cells (Figure 7B). Relative to [S] cells, [R] cells up‐regulated double‐strand break repair (DSB) pathway, especially the high‐fidelity homologous recombination DNA‐repair (HRR) pathway, concomitantly down‐regulated error‐prone nonhomologous end joining (NHEJ) signaling (Figure S6B, Supporting Information). Thereinto, transcriptional expression of HRR genes BRCA1, BRCA2, RAD51 and PALB2 in [R] cells was observed significantly up‐regulated compared to budding RTP cells ([B]) (Figure S6C, Supporting Information). Pseudotime analysis utilizing Monocle2 allowed us to reconstruct the differentiation continuum of individual cells according to their transcriptional similarities.^[^ ^27^ ^]^ Cells from five stages were organized into one main bifurcate (Figure 7C). Distribution of late progenies coincided with early repopulated cells, which turned back to the right branch overlapping with unirradiated state. Deletion of p53 had no influence on cellular distribution (Figure S6D). Comparison of repopulated cells (D50R) versus untreated cells in tumorigenicity of nude mice and clone formation after irradiation revealed no significant difference (Figure 7D,E). In sum, data‐above suggested the restoration of repopulating cells and its possible participation in tumor repopulation.

### 2.8 Clinical Value of the Budding RTP Gene Signature

We sought to determine whether the budding RTP gene signature that enables cancer cells to persist and repopulate following radiation can also be enriched in patient tumors after radiotherapy. RT‐PCR detection of budding RTP gene‐expressions in PDX model post two times 8 Gy irradiation revealed that most RTP gene levels (CST3, CDC37, IGFBP6, ISYNA1, RN7SL2) increased on day 9 when RTP cells were significantly enriched and partial fell back on day 82 when tumor repopulated (Figure 7E). There were also budding RTP genes like SNCG and RPS12 displaying enhancement of expression on day 82, where they were functioning to generate daughter cells. We next examined the budding RTP signature expression in residual rectal cancer cells post neoadjuvant chemo‐radiotherapy (nCRT) collected from published transcriptomes of 52 patients with locally advanced rectal cancer (LARC, GSE94104^[^ ^28^ ^]^ and GSE190826^[^ ^29^ ^]^), as compared with their matched diagnostic specimens. Results showed that budding RTP signature computed by ssGSEA (Figure 7G) and expression of [B]_type genes (Figure 7H; Figure S6E, Supporting Information) after nCRT treatment were significantly higher than that of before treatment. Upregulation of RTP gene markers (SNCG, CST3 and RPS12) was also found in three resected tumor samples we collected from pPR (pathological partial response) LARC patients after receiving nCRT, which was coherent with the poor regression grade of these patients (Figure 7I). Collectively, these data suggest that our budding‐RTP signature can define residual tumor of patients treated with radiotherapy.

To explore clinical significance of gene signatures of RTP cells, we used database of The Cancer Genome Atlas (TCGA) to evaluate whether budding DEGs could predict oncotherapy prognosis. A K‐Top‐Scoring‐Pair (KTSP) method^[^ ^30^ ^]^ was adopted to train a binary classifier correlating gene expression with clinical response in patients who merely received radiotherapy. When using the simplified 11 pair‐wise TSPs genes (TSPs) to evaluate radio‐sensitivity, seven points or more were considered as “sensitive”; otherwise, defined as “resistant” (Figure S6F, Supporting Information). Consistently, overall survival (OS) of the expected sensitive group was significantly higher than resistant group (Figure 7J). The 11TSPs classifier was equally accurate in predicting OS of patients received concurrent chemo‐radiotherapy even chemotherapy (Figure 7G; Figure S6G, Supporting Information). Regression model analysis reflected good classification ability of 11TSPs for survival prognosis of patients received oncotherapy (Figure S6 H,I, Supporting Information). We next applied 11TSPs to 6 LARC patients and results showed that 11TSPs achieved the sensitivity of 83.3% in predicting sensitive response to chemo‐radiation (Figure 7I; Figure S6J, Supporting Information). We also extended our KTSP model with transcriptomes derived from locoregionally advanced nasopharyngeal cancer patients with or without metastasis after radical radiotherapy,^[^ ^31^ ^]^ or paired glioblastoma PDXs with acquired radio‐resistance and untreated radio‐sensitive status.^[^ ^32^ ^]^ KTSP scores of group with good prognosis (non‐metastasis or radio‐sensitive) are significantly higher than group with poor prognosis (metastasis or acquired radio‐resistance) (Figure S6I, Supporting Information), demonstrating good accuracy of 11‐paired TSPs in predicting prognosis of cancer patients with radiotherapy.

## 3 Discussion

Tumor cell repopulation is a major cause for radiation failure and the underlying mechanism remains to be illustrated. Our study suggested a reversible radiation‐tolerant state with process of tumor cell poly‐ and then de‐polyploidization in response to radiation. These poly‐aneuploid RTP cells exhibit a distinct phenotype characterized by enlargement of cell sizes and nucleus, complex karyotype, cell cycle arrest and engagement of quiescence, and a gene signature represented by CST3, which might support survival maintenance of RTP cells. Moreover, RTP cells accomplished their de‐polyploidization through a process of virus‐like DNA assembly then budding off proliferating progenies with lipid bilayer envelope, accompanied with specific expression of a gene signature represented by SNCG. These two gene signatures could provide markers to help identify the small fractions of RTP cells progressing toward viral‐mimicry budding after radiotherapy. As shown in this study, loss of functions of either CST3 or SNCG could curtail the budding of RTP cells and prevent tumor repopulation, which provide an attractive therapeutic opportunity for sensitizing radiotherapy.

Phenomenon of drug‐tolerant persister (DTP) is most commonly reported in response to targeted drugs, which function as tumor suppressor by targeting growth‐associated proteins on cell membrane or in the cytoplasm. DTP enables cancer cells to withstand the short‐term stressful stimuli and its slow‐cycling state facilitates metabolic changes and other mechanisms to allow further adjustment.^[^ ^33^ ^]^ Increasing evidence established the key role of non‐mutational mechanisms underlying the slow‐cycling phenotype of residual persister cells and their reversibility to a non‐persister state.^[^ ^34^ ^]^ Upregulation of histone H3 lysine demethylase KDMs has been observed in DTP states of various tumor models.^[^ ^5^, ^35^ ^]^ Activation of KDMs is associated with repressed chromatin state of DTP. Knockdown of KDM5A or disruption of the heterochromatin formation mediated by H3K9me3 reduced survival of persister cells,^[^ ^5^, ^36^ ^]^ suggesting the dependence of drug tolerance on chromatin remodeling. Different from DTP, irradiation yields breakage of double‐strand DNA, accompanied with severe damage to cellular structure. Although with the similarities that both RTP cells and DTP cells possess the ability to survive the damage of cytotoxic stress and escape from dormancy to re‐enter into cell cycling, complex karyotype arose with severely damaged RTP cells renders it unable to undergo mitosis.

Akin to the cellular hypertrophy via polyploidization in acute organ failure,^[^ ^9^, ^37^ ^]^ poly‐aneuploidy might endow cancer cells with greater capacity and better fitness against genotoxic stress than the diploid state.^[^ ^38^ ^]^ On the one hand, our study implicated that polyploidization of RTP cells is an important manner through which cancer cells could weather the storm of radiation‐induced DNA damage. On the other hand, given that massive aneuploidy and chromosome instability will bring on lethal genomic chaos, polyploidization may be a temporarily adaptive strategy.^[^ ^39^ ^]^ Results from our study showed that depolyploidization of RTP cells enabled relatively normal genome, similar to their unirradiated parental cells, to be re‐assembled into a bud. A similar process named “neosis” was first posited by Rajaraman about 20 years ago. “Neosis” described multinucleated cancer cells generating mononuclear offspring through a rare asymmetrical cell division initiated by nuclear budding and followed by cytokinesis.^[^ ^40^ ^]^ Here, we highlighted the karyotype reversibility from poly‐aneuploid RTP cells to repopulated cells. And the way of progenies production is highly reminiscent of production of virus with envelope. Exploiting the host synthesis machinery, viruses replicate their genomic materials in multiple copies, which can be precisely packaged into new virions to be released. In budding of poly‐aneuploid RTP cells, chromosomes that are almost identical to the parental cells are observed likewise assigned correctly to the progeny cells. That virion‐releasing signature associated with membrane abscission and subsequent release of repopulating daughter cells was indeed observed in budding RTP cells. Even if the first assignment is not correct, budded cells will actively initiate DNA repair mechanisms, and chromosomes (including structure and number) in the repopulated cell are verified to get closer to that of the parent cells.

“Stranger and danger” mode is widely acknowledged upon interferon induction.^[^ ^41^ ^]^ Similar to “stranger” recognition by pathogen‐associated molecular pattern during viral infection, endoreplicated cells induced by radiation can release nucleic acids into cytoplasm. These nucleic acids then serve as “danger” signal to activate canonical IFN‐mediated antivirus signaling under a sterile condition.^[^ ^42^ ^]^ In our data, levels of dsRNA as well as the sensor RIG‐I receptor showed significant increases in RTP cells. In addition, both pre‐budding RTP cells and budding RTP cells had significantly elevated IFN‐I production and activated IFN signal pathway despite with different level. Similar response was observed in HIV‐latently infected host cells as well as in HIV‐producing cells when compared with uninfected cells.^[^ ^43^ ^]^ Perhaps, IFN production and IFN signal pathway activation are an evolutionarily conserved mechanism related to survival fitness under stress. Our findings, that exposure of early RTP cells to additional IFN‐I significantly boosted the host antiviral effects and precluded RTP budding‐involved tumor repopulation, might represent a promising therapeutic approach to prevent tumor relapse.

RTP cells share a similarity with CSCs in that they are associated with radio‐resistance and repopulation of cancer cells. Rather than enrichment in conventional stemness signatures, we observed diapause‐ and RSC‐like phenotype of budding RTP cells, which are similarly triggered by stress and function to support regeneration. Consistently, in recurrence of acute myeloid leukemia, residual cancer cells also enter a senescence‐like status, mimicking diapause‐like signature independent of stemness signatures to survive chemotherapy and repopulate the disease later.^[^ ^21b^ ^]^ It is in fact through viral‐budding like division of RTP cells to re‐propagate tumor, rather than the asymmetric self‐renewal of stem cells to perpetuate itself. RTP is thus “a bad seed” in tumor repopulation after radiotherapy as a more CSC‐like state but not overlap with other stem cell properties.

In this study, we identified two gene panels represented by CST3 and SNCG along with the formation and the budding of RTP cells. Cystatin‐C encoded by CST3 is a cytokine ubiquitously expressed in most cell types. Elevation of cystatin C has been proposed as a potential marker for early detection of kidney injury, and also been reported as protective factor in injured cardiomyocytes^[^ ^44^ ^]^ as well as nervous system in response to stress.^[^ ^45^ ^]^ Mechanisms through which cystatin‐C mediates stress responses might include its protease‐inhibitory function, regulation of cell proliferation, induction of cell autophagy and modulation in inflammatory response.^[^ ^45^, ^46^ ^]^ Interestingly, hypertrophy through polyploidization is a critical strategy in kidney and heart to sustain organ function after acute failure.^[^ ^37^ ^]^ Although our study unveiled that increased expression of CST3 is highly involved in cellular polyploidization to maintain survival, related high‐quality studies on the topic is scarce. The role of CST3 in stress‐induced polyploidization and the detailed molecular mechanisms merit the follow‐up work.

*γ*‐synuclein (SNCG), together with *α*‐ and *β*‐synuclein, constitutes the highly conserved synuclein family and participates in development of malignant and neurodegenerative diseases. Expression level of SNCG is positively relevant to tumor progression across various types of cancers.^[^ ^47^ ^]^ And elevated expression of SNCG confers resistance to anti‐cancer treatment.^[^ ^48^ ^]^ As a co‐chaperone, SNCG could interact with multiple kinases in cytotoxic situation, increasing stability of Akt and activating mitogen‐active protein kinase pathways,^[^ ^49^ ^]^ or regulating microtubule by acting with mitotic checkpoint kinase BubR1.^[^ ^50^ ^]^ Our results reveal the close relationship between highly expressed SNCG and budding of irradiation‐induced RTP cells. The specific molecular mechanisms remain unclear, but it could be one possibility that, as an amyloidogenic protein induced by stress, when *γ*‐synuclein is propagated from parental RTP cell to budded progeny cells, cytoplasmic stress on forthcoming is synchronously transmitted to progenies in a prion‐like manner.^[^ ^51^ ^]^

Our study revealed a novel mechanism of tumor repopulation, i.e., radiation‐induced RTP cells produce progenies via a viral budding‐like division. Targeting the radiation‐persistent cells with budding potential might be a therapeutic approach to eliminate the minimal residual disease. Besides, 11TSPs classifier based on the budding gene set also offer prognostic stratification to pan‐cancer patients who receive cytotoxic oncotherapy, favoring the early identification of individuals with refractory disease.

## 4 Experimental Section

### Cell Culture and Irradiation

Human colorectal cancer cells HCT116, Caco‐2, breast cancer cells MDA‐MB‐231, non‐small cell lung cancer cells A549, prostatic stromal myofibroblast cells WPMY1, and HEK293FT were purchased from the Cell Bank of the Chinese Academy of Sciences (Shanghai, China). HCT116 p53−/− (379.2) and p53+/+ (4016) were homologous recombinant cell lines as a gift from the laboratory of Dr. Bert Vogelstein.^[^ ^52^ ^]^ Cells were cultured in Dulbecco's MEM medium containing 10% (v/v) FBS, 1% penicillin‐streptomycin (all from Life Technologies) at 37 °C in a 5% CO_2_ atmosphere. For subculture of suspended HCT116, cells were centrifuged at 1000 rpm for 3 min and then counted to reseed at a proper density (ultra‐low attachment 6‐well plates, 10^5^–10^6^ cells per well). All cell cultures were regularly tested for *Mycoplasma* contamination. Irradiation of X‐ray (dose rate 300 cGy min^−1^) was delivered to cells using a Varian Clinaci X linear accelerator (Varian Medical Systems Inc.).

### Collection of Giant and Normal‐Sized Cells

Irradiated cells were trypsinized to generate single‐cell suspensions on day 6 after 8 Gy treatment. Normal‐sized cells were separated by filtering the cellular mixture sequentially through 20 and 10 µm pore‐sized nylon meshes (Spectrum Laboratories, Inc.), while giant cells were retained on the top of 20 µm meshes and harvested by several rinses with the complete medium.^[^ ^53^ ^]^

### Mice Experiments

All animal studies were conducted in accordance with the ethical standards of the Animal Care and Use Committee of Shanghai General Hospital, Shanghai Jiao Tong University School of Medicine, China (No. 2019‐A020‐01). For tumor xenografts of HCT116, BALB/c nude mice (4–6 weeks old) were utilized for the subcutaneous tumor model. A total of 5 × 10^6^ cells in 100 µL PBS were injected subcutaneously into the left flank of mice. Tumors were exposed to 10 Gy X‐ray (dose rate 300 cGy min^−1^) when they reached 250 mm^3^. Following radiation, mice were sacrificed when the tumor size reached 2000 mm^3^ or the mice exhibited cachexy.

For construction of the patient‐derived xenograft (PDX) model, tumor gross excised from a rectal adenocarcinoma patient was cut into small fragments and then implanted subcutaneously into NOD/SCID mice according to the previous study. For further maintenance, PDX tissues were cut into small fragments and injected subcutaneously into the left flank of 6‐week‐old male BALB/c nude mice. When the average tumor diameter reached 8 mm, mice were randomly separated into control groups or subjected to two times 8 Gy‐fractionated radiation (dose rate 300 cGy min^−1^). Mice were sacrificed if the tumor size reached 2000 mm^3^.

For intravenous metastasis formation assay, giant, normal‐sized and mixture cells were first collected. A total of 2.5 × 10^5^ giant, normal‐sized or mixture cells in 50 µL PBS were intravenously injected into the vein of BALB/c mice, respectively. Body weights of mice were measured twice a week. After 82 days, mice were sacrificed and lungs were then harvested for H&E and anti‐Pan Actin staining. Metastasis foci of more than 50 Pan Actin‐stained positive cells were counted and analyzed.

For the in vivo LDA assay, primary tumors from 10Gy‐treated HCT116 xenografts were dissociated into a single‐cell suspension on day 8 after irradiation, and then giant (G8) and normal‐sized cells (N8) were, respectively, selected by meshes. A primary tumor from untreated HCT116 xenograft was also dissociated into single cells (NC). For the simplified LDA assay, 8Gy‐irradaited HCT116 cells were cultured for 6 days and isolated to giant (G6) and normal‐sized cells (N6). Untreated HCT116 cells were also trypsinized into single cells (NC). The isolated cells were next diluted serially, resuspended in 0.1 mL 50% Ceturegel Matrix LDEV‐Free (Yeasen Biotechnology) diluted in PBS, and injected subcutaneously in the right flank of the nude mice at desired cell doses, i.e., 1 × 10^6^, 1 × 10^5^ and 1 × 10^4^ donor cells per mouse. Tumor incidence was monitored. Mice weight and tumor growth were measured every 3 days post inoculation. CSC frequency was calculated by ELDA software.^[^ ^54^ ^]^

### Histopathological Analysis

Tumor tissues were fixed in 10% neutral‐buffered formalin, embedded in paraffin and serially cut into 4 µm sections for H&E staining and IHC staining as previously described.^[^ ^55^ ^]^ Tissue sections were stained with hematoxylin and eosin, anti‐Ki67 (1:200, CST 9027), anti‐CST3 (1:100, Santa Cruz Biotechnology sc‐515732), anti‐SNCG (1:100, Abcam 55 424), anti‐human pan Actin (1:1200, CST 8456), or anti‐CD44v6 (1:300, R&D systems) primary antibody following the manufacturer's instructions.

### Collection of Patient Samples

Formalin fixed, paraffin‐embedded (FFPE) patients specimens from 6 LARC patients were obtained from the Shanghai General Hospital. Approval for this study was required from the Ethics Committee of the Shanghai General Hospital (2016KY130). Patients with tumors clinically staged II‐III (cT3/4 and/ or N+) received neoadjuvant chemo‐radiotherapy (CRT) (Table S7, Supporting Information). Long‐course radiotherapy consisted of 50–53 Gy total in 25 fractions and concurrent chemotherapy were administrated based on Capecitabine. Surgery was performed 4 weeks after completion of CRT. Pre‐CRT biopsy samples (total of 6) and available post‐CRT resection specimens (total of 6) were, respectively, obtained from colorectoscopy and surgery. Only 3 out of 6 post‐CRT specimens had some visible tumor tissue left. The clinical responses were evaluated by the Response Evaluation Criteria in Solid Tumors (RECIST). Tumor regression grades were assessed according to 8th edition of American Joint Commission on Cancer (AJCC) guideline.

### CRISPR‐Mediated Gene Knockout and Lentiviral Infection

Genes‐knockout cell lines were generated by means of CRISPR‐cas9 technology as previously reported.^[^ ^56^ ^]^ Single guided RNA (sgRNA) primers targeting the gene *SNCG* (Gene ID: 6623), and *CST3* (Gene ID: 1471) were designed using the online tool (https://portals.broadinstitute.org/gpp/public/analysis‐tools/sgrna‐design).^[^ ^57^ ^]^ The sgRNA target sequences are listed in Table S2 (Supporting Information). Annealed primers were cloned into the plasmid LentiCRISPR v2 (gifts from Dr. Feng Zhang through Addgene, plasmid 52 961).

The constructed CRISPR lentivirus vectors were then transduced into HEK293T cells together with the packaging plasmids (pSPAX2 and pMD2.G) using Lipofectamine 2000 Transfection Reagent (Invitrogen) according to the manufacturer's protocol. The supernatant containing viruses were harvested at 48 hours after transfection and then utilized to infect cells through a 0.44 µm filter. Puromycin (1 µg mL^−1^) was added to select the positively infected cells for 2 weeks. Then these infected cells were seeded into 96‐well plate with one cell per well. Single colonies were amplified and validated by western blot. The clones with no detectable target signal were kept for subsequent experiments.

### Immunofluorescence Staining (IF) and Immunofluorescence In Situ Hybridization (iFISH)

For cellular IF analysis, cells upon different treatments were cultured on cover glasses (Fisher Scientific) coated on the bottom of 24‐well plates. At indicated time, cells were fixed with 4% paraformaldehyde for 15 min, permeabilized with 0.3% Triton X‐100 for 15 min and blocked with blocking buffer containing 5% bovine serum for 1 h. Fixed cells were subsequently incubated with the primary antibodies overnight at 4 °C, followed by incubation with corresponding Alexa Fluor 594, or 488‐conjugate secondary antibodies (CST) at room temperature for 1 h. Primary antibodies used as follows: anti‐CST3 (Santa Cruz Biotechnology, sc‐515732), anti‐SNCG (Abcam, 55 424), anti‐*γ*‐tubulin (Abcam,11 317), anti‐RIG‐I (sc‐376845), anti‐IFN‐*α* (sc‐373757), anti‐ISYNA1 (Abclonal, A8965), anti‐CHMP2A (affinity,DF12147), anti‐VPS28 (sc‐166537), anti‐CD44v6 (R&D, BBA13), anti‐CD44 (CST,5640), and anti‐CD133 (Proteintech, 18470). Filamentous actin (F‐actin) was stained with phalloidine (Yeasen) as needed. Cell nuclei were accomplished with DAPI (Life Technologies). Slides were mounted with mounting medium (Vector Laboratories) and sealed for visualization with a confocal scanning microscope (Leica) or fluorescence microscope (Leica).

For IF and iFISH co‐staining, iFISH Human CTC Identification Kit (Cytelligen) was used grounding on the manufacturer's instructions with the following alterations. After labeled with primary and appropriate secondary antibody as described above in IF, cells fixed on the coated slides were subjected to Vysis Centromere Probe CEP8 (or CEP12) Spectrum Orange (or FITC, all from Abbott Laboratories) and sealed for hybridization for 3 h using the S500 Stat Spin Thermo Brite Slide Hybridization System (Abbott Molecular). Images were captured using Zeiss Axio Imager Z2 microscope. When using suspended cells for iFISH, samples were centrifuged and washed by cleaning buffer, followed by spreading onto a formatted slide overnight at 30 °C. Hybridization with centromere probe was the same as described above, and subsequent incubation with Alexa Fluor 555‐conjugated anti‐CK18 antibody (Cytelligen) was additionally performed. The remaining steps were repeated as above.

### SMART RNA‐Sequencing

Cellular samples for SMART RNA‐seq includes untreated cells ([N] stage), RTP cells prior to budding in the first week post‐8 Gy ([P] stage), budding RTP cells ([B] stage) and the newly budded small cells ([S] stages) as well as repopulating cells on day 30 after 8 Gy treatment ([R] stage). The tumor cells were first minced with PBS consisting of 2 U µL^−1^ RNAase inhibitor. Under a microscope, single cells in individual stages were selected and aspirated with pipettes. The selected cells were immediately added lysis buffer for single‐cell RNA extraction, reverse transcription and cDNA amplification using Single Cell Full Length mRNA‐Amplification Kit (Vazyme, N712) per manufacturer's protocol. RNA‐seq libraries were constructed from purified cDNA using TruePrep DNA Library Prep Kit V2 for Illumina (Vazyme, TD503) according to the manufacturer's instruction. cDNA libraries were then sequenced on an IlluminaNovaSeq 6000.

### Single‐Cell RNA‐Sequencing

Cells at stage prior to budding, when budding occurs and after budding were prepared for 10× scRNA‐seq and two samples named “Mix1” and “Mix2” were loaded onto an independent 10×Chrominum instrument (10× Genomics) per the user instructions. In brief, 8Gy‐irradiated HCT116 cells in different stages constituting group IR were collected for “Mix1”, followed by the assessment of cellular viability and adjustment of concentration to 1000 µL^−1^. Before the same process of preparation for “Mix2”, group of NC, IR and R cells were incubated with individual antibody tags (Biolegent TotalSeq) to be distinguishable from each other. An input of estimated 10000 cells was transfer to each channel. Single‐cell libraries were constructed using the Single Cell 3’ Reagent Kit (10× Genomics, v3.1). Libraries were then performed quality control using Bioanalyzer High Sensitivity DNA kit (Agilent) and sequenced on an Illumina NextSeq 500 platform (Genergy Inc.).

### Bulk‐RNA Sequencing

At least 10^6^ giant and small HCT116 cells sorted on day 6 post 8 Gy, as well as untreated HCT116 cells were prepared for RNA‐seq of Figure 2C. Giant HCT116 cells isolated on day 6 after 8 Gy, with the treatment group applied with 0.1 ng mL^−1^ (IFN‐L) or 10 ng mL^−1^ IFN*α*2b (IFN‐H) for 1 day, were harvest on day 8 and prepared for RNA‐seq of Figure 4D. Total RNA was extracted using RNeasy kit (QIAGEN, 74 106) according to the manufacturer's protocol and quantified using NanoDrop Spectrophotometer (Thermo Scientific). RNA libraries were prepared using TruSeq Stranded mRNA Library Prep Kit for NeoPrep per the standard protocols and sequencing was performed using the Illumina HiSeq 2500 high‐throughput sequencing system.

### Statistical Analysis

Data were performed using GraphPad Prism 8 Software (Graphpad Software) and SPSS statistical software 13.0.1. Comparison between two groups was conducted by using Student's two‐sided *t*‐test. Multiple group comparisons were performed using one‐way or two‐way ANOVA test (involving two variables). *p*<0.05 was considered as significant.

## Conflict of Interest

The authors declare no conflict of interest.

## Supporting information

Supporting Information

Supplemental Table 1–4

Supplemental Table 5

Supplemental Table 6

Supplemental Table 7

## Data Availability

The data that support the findings of this study are available in the supplementary material of this article.
