# Supplementary material for: Cancer Cells Enter an Adaptive Persistence to Survive Radiotherapy and Repopulate Tumor
Source: Adv Sci (Weinh). 2023 Jan 19;10(8):2204177. doi: 10.1002/advs.202204177 (PMC10015890; doi:10.1002/advs.202204177)
Supplement: Supplementary file 1 — Supporting Information [file ADVS-10-2204177-s002.pdf]

Supplemental figures

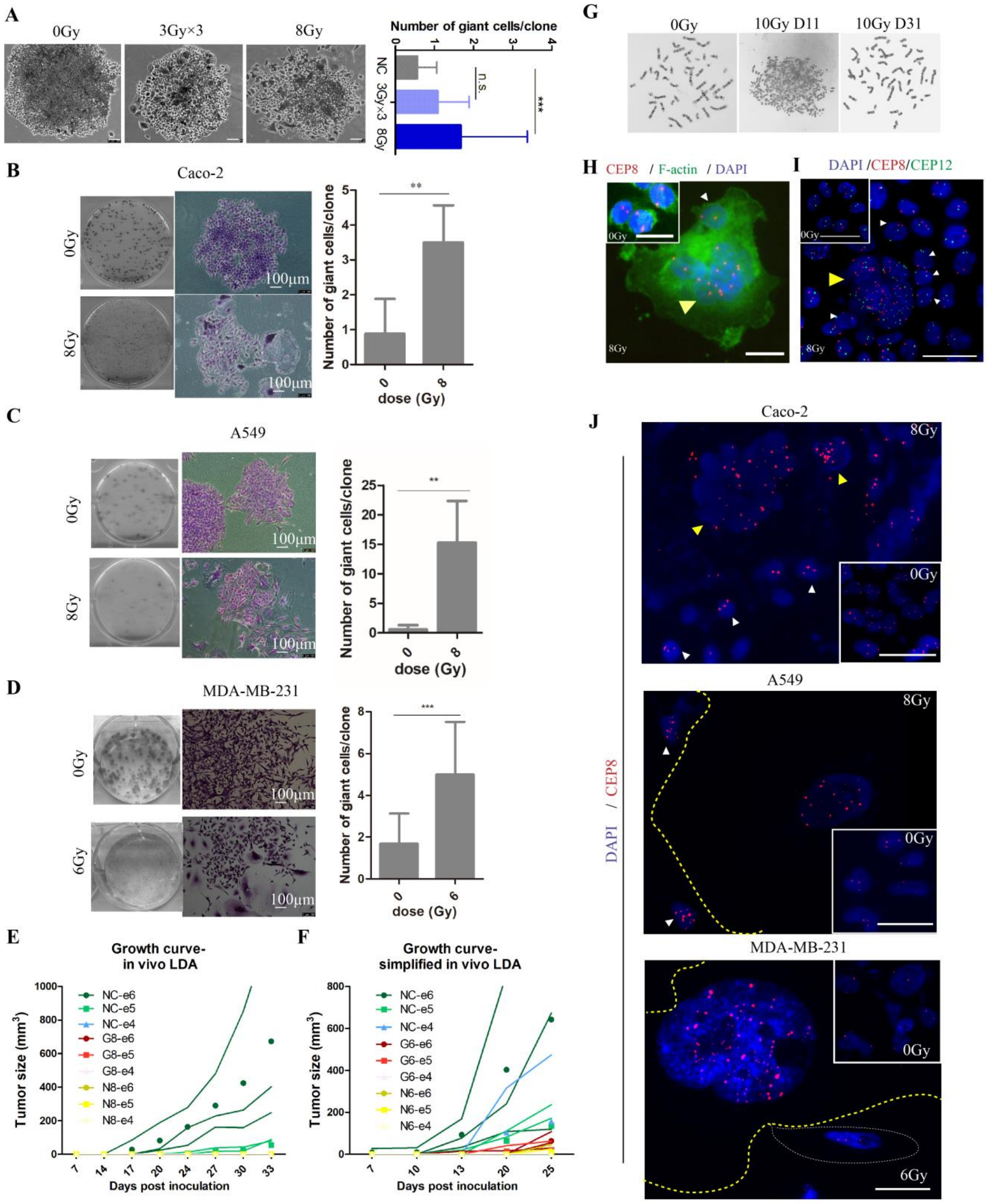

Figure S1. Poly-aneuploid RTP cells are capable of repopulating tumor *via* virus-like budding division.

A-D) Giant cancer cells (>3 times the mean area pre-irradiation) were predominantly found in irradiation-treated colonies of HCT116, Caco-2, A549 and MDA-MB-231 cells. Numbers of giant cells per colony were plotted; one-way ANOVA for (A) and student's *t* test for (B-D).
E-F) Growth curve of *in vivo* limited dilution assays (LDA) from primary tumor-derived cells and simplified LDA using *in vitro* irradiated tumor cells.
G) Karyotype analysis of corresponding cells in **Figure 3B** reflecting the copy number variation of overall chromosomes.
H-I) Representative immunofluorescent images of budding RTP cells (yellow triangle), which were surrounded by diploid normal-sized cells (white triangle). Ploidy number, CEP8 (red); cytoskeleton, F-actin (green) or CEP12 (green). Scale bars, 25 μm.
J) 8Gy-induced RTP cells of Caco-2, A549 or MDA-MB-231 are producing daughter cells with relative normal CEP8 number (yellow dotted lines outline RTP cells; white triangles or lines denote progenies). Inset white boxes show representative iFISH images of untreated cells stained by CEP8. Scale bars, 25 μm.

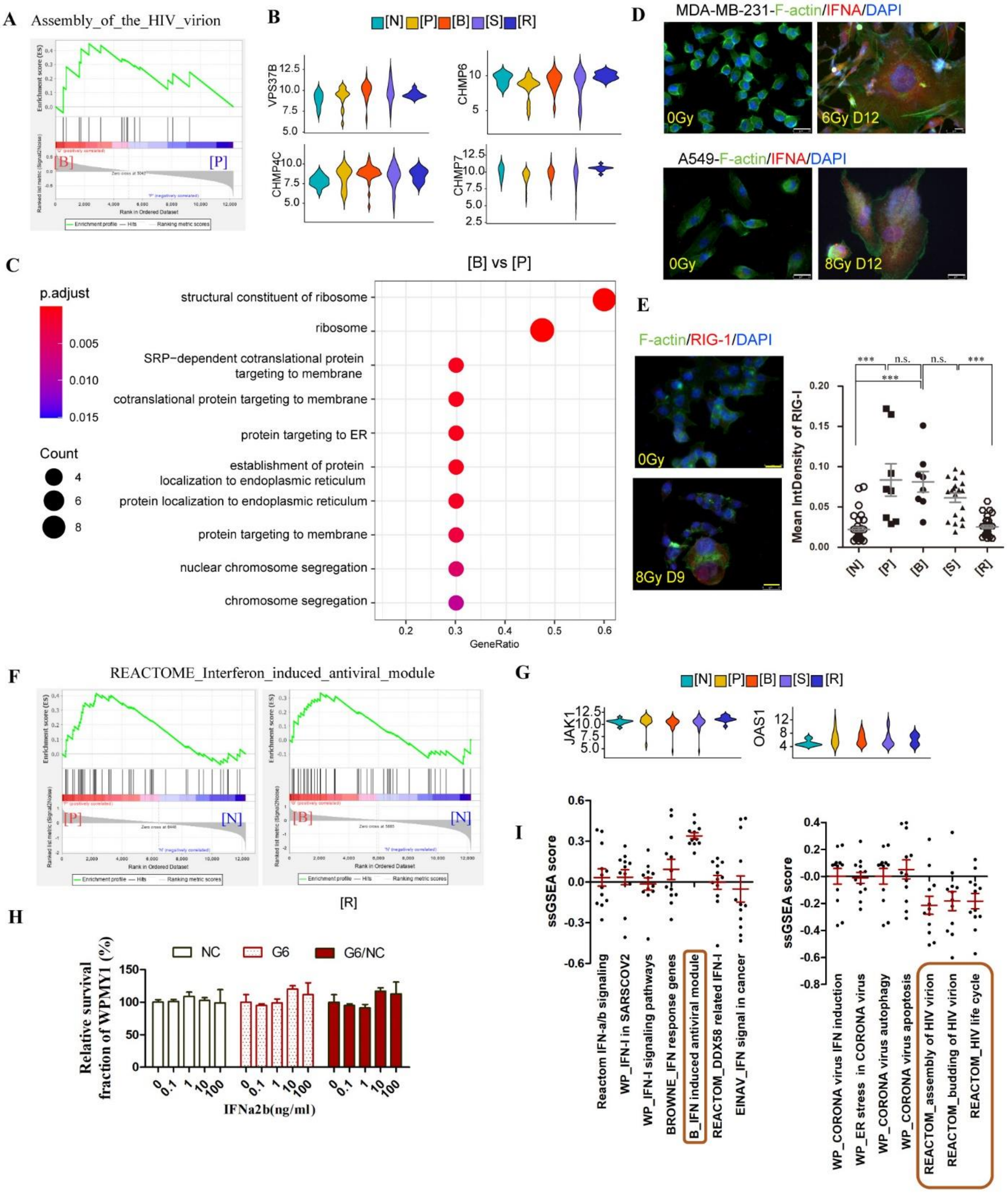

A Assembly_of_the_HIV_virion
[B]
[P]
B
[N] [P] [B] [S] [R]
VPS37B
CHMP6
CHMP4C
CHMP7
C
[B] vs [P]
p.adjust
0.005
0.010
0.015
Count
4
6
8
structural constituent of ribosome
ribosome
SRP−dependent cotranslational protein targeting to membrane
cotranslational protein targeting to membrane
protein targeting to ER
establishment of protein localization to endoplasmic reticulum
protein localization to endoplasmic reticulum
protein targeting to membrane
nuclear chromosome segregation
chromosome segregation
GeneRatio
D
MDA-MB-231-F-actin/IFNA/DAPI
0Gy
6Gy D12
A549-F-actin/IFNA/DAPI
0Gy
8Gy D12
E
F-actin/RIG-1/DAPI
0Gy
8Gy D9
Mean IntDensity of RIG-I
n.s.
[N] [P] [B] [S] [R]
F
REACTOME_Interferon_induced_antiviral_module
[P]
[N]
[B]
[N]
[R]
G
[N] [P] [B] [S] [R]
JAK1
OAS1
H
NC
G6
G6/NC
Relative survival fraction of WPMY1 (%)
IFNa2b(ng/ml)
I
ssGSEA score
Reactom IFN-a/b signaling
WP_IFN-I in SARSCOV2
WP_IFN-I signaling pathways
BROWNE_IFN response genes
B_IFN induced antiviral module
REACTOM_DDX58 related IFN-I
EINAV_IFN signal in cancer
WP_CORONA virus IFN induction
WP_ER stress in CORONA virus
WP_CORONA virus autophagy
WP_CORONA virus apoptosis
REACTOM_assembly of HIV virion
REACTOM_budding of HIV virion
REACTOM_HIV life cycle

Figure S2. Type-I interferon mediated anti-viral signaling suppresses budding of RTP cells.

A) Positive enrichment for assembly of HIV virion in budding RTP cells ([B]) comparing with pre-budding RTP cells ([P]).

B) Violin plots showing expression of endosomal sorting complex required for transport (ESCRT) proteins: VPS37B, CHMP4C, 6 and 7.

C) Bubble plot showing pathways enriched in [B] *vs* [P].

D) Protein expression of IFNA (red) and F-actin (green) in untreated and 8Gy-treated budding MDA-MB-231 and A549 RTP cells by fluorescence staining. Scale bar, 25 mm.

E) Expression of RIG-I (red) in untreated and 8Gy-induced budding HCT116 RTP cells. Mean RIG-I fluorescence intensity of the indicated cells were shown at right. Scale bar, 25 mm.

F) Gene set enrichment analysis (GSEA) showing positive enrichment for interferon-I induced antiviral module in pre-budding ([P]) and budding RTP cells ([B]) relative to untreated cells ([N]).

G) Violin plot showing dynamic expression of interferon-stimulated genes (ISGs): JAK1 and OAS1.

H) Effect of IFNa2b on survival fraction of WPMY1 cells.

I) ssGSEA score of 12 residual tumor models after drug-treatment *versus* their untreated baseline in enrichment of gene signatures related to type-I IFN (left panel) and virus life cycle (right panel).

[N], untreated cells; [P], pre-budding RTP cells post 8Gy; [B], budding RTP cells of day 9 post 8Gy; [S], budded progeny cells of day 9 post 8Gy. *p < 0.05, **p < 0.01, ***p < 0.001; n.s., not significant.

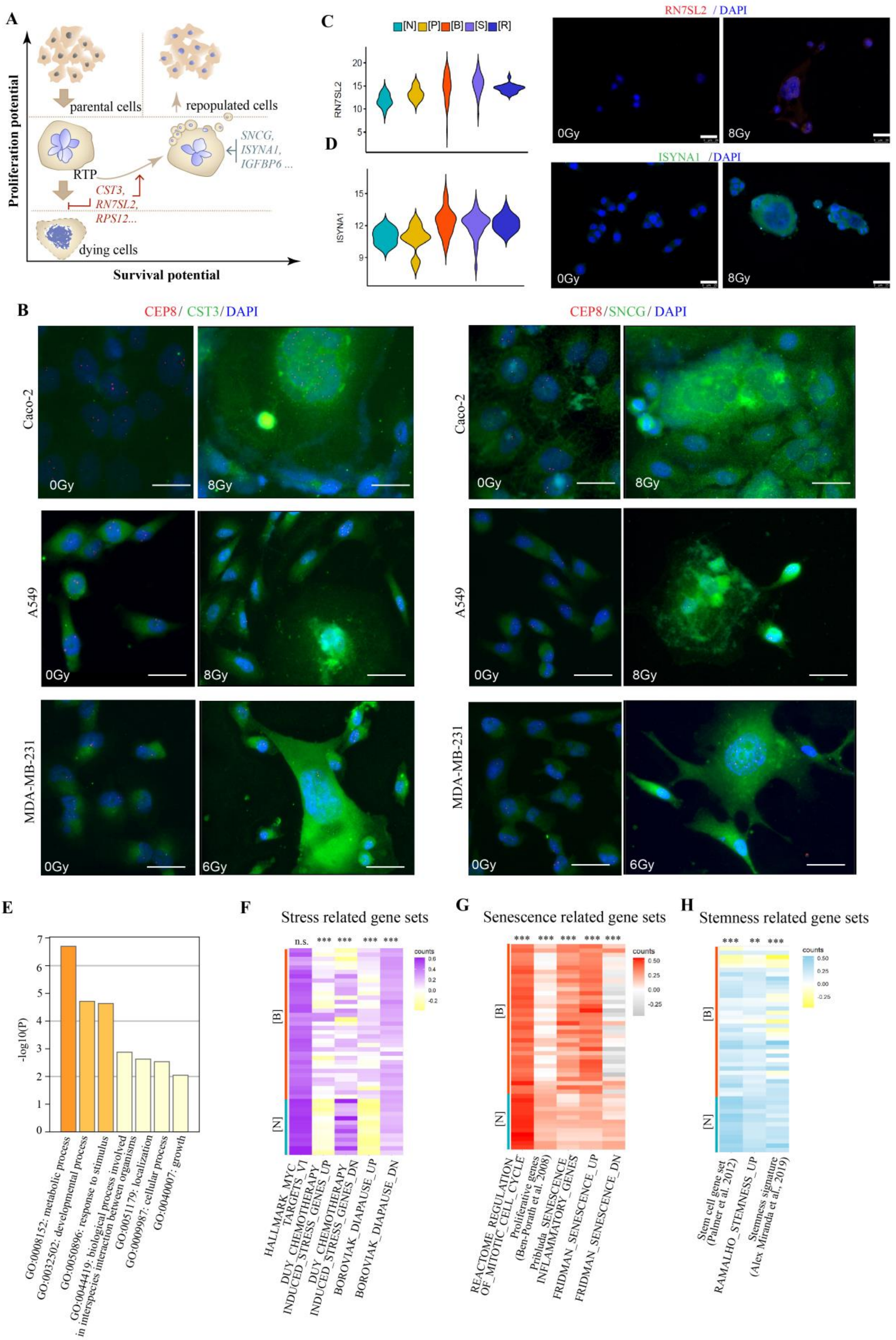
A
Proliferation potential
parental cells
repopulated cells
SNCG,
ISYNA1,
IGFBP6 ...
RTP
CST3,
RN7SL2,
RPS12...
dying cells
Survival potential
C
[N] [P] [B] [S] [R]
RN7SL2
D
ISYNA1
RN7SL2 / DAPI
ISYNA1 /DAPI
0Gy
8Gy
B
CEP8/ CST3/ DAPI
CEP8/ SNCG/ DAPI
Caco-2
A549
MDA-MB-231
0Gy
8Gy
6Gy
E
-log10(P)
GO:0008152: metabolic process
GO:0032502: developmental process
GO:0050896: response to stimulus
GO:0044419: biological process involved in interspecies interaction between organisms
GO:0051179: localization
GO:0009987: cellular process
GO:0040007: growth
F
Stress related gene sets
n.s. *** *** *** ***
counts
[B]
[N]
HALLMARK_MYC_TARGETS_V1
DUY_CHEMOTHERAPY_INDUCED_STRESS_GENES_UP
DUY_CHEMOTHERAPY_INDUCED_STRESS_GENES_DN
BOROVIAK_DIAPAUSE_UP
BOROVIAK_DIAPAUSE_DN
G
Senescence related gene sets
*** *** *** *** ***
REACTOME_REGULATION_OF_MITOTIC_CELL_CYCLE
Proliferative genes (Ben-Porath et al. 2008)
Pribluda_SENESCENCE_INFLAMMATORY_GENES
FRIDMAN_SENESCENCE_UP
FRIDMAN_SENESCENCE_DN
H
Stemness related gene sets
*** ** ***
Stem cell gene set (Palmer et al. 2012)
RAMALHO_STEMNESS_UP
Stemness signature (Alex Miranda et al., 2019)

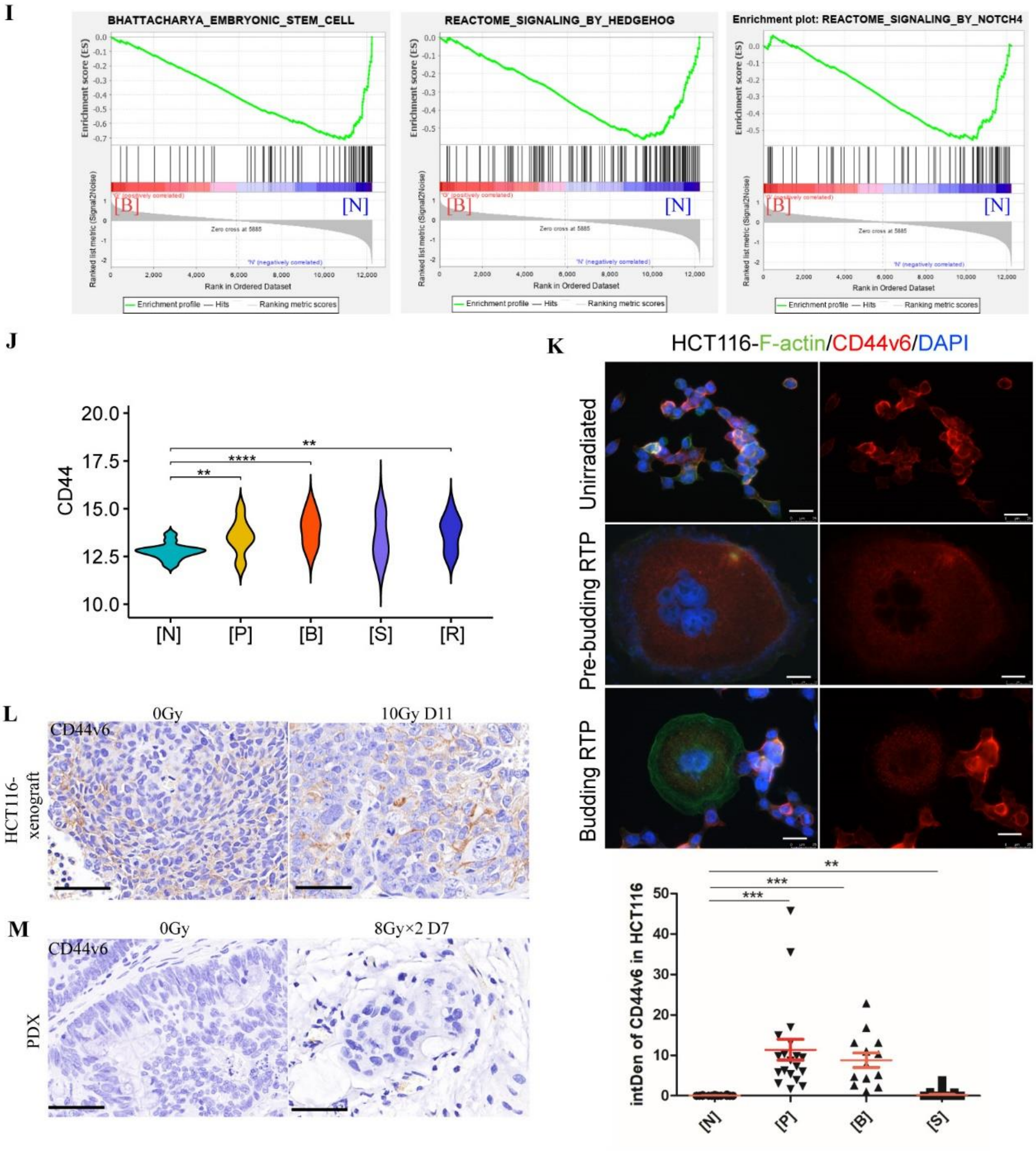

Figure S3. Transcriptomic signatures depicting budding of RTP cells.

A) Summary schema showing roles of two budding-associated transcriptomic signatures in tumor repopulation following irradiation. The first panel is associated with survival of giant RTP cells, while the second panel mediates budding of RTP cells.

B) Typical expression of CST3 (left panel) and SNCG (right panel) in untreated and 8Gy-

induced budding RTP cells of Caco-2, A549 and MDA-MB-231 by immunofluorescence staining. Scale bar, 25mm.

C-D) Transcriptional and protein expression of RN7SL2 (matching the first panel) and ISYNA1 (matching the budding panel) in untreated and 8Gy-induced budding RTP cells of HCT116 *in vitro*. Scale bar, 25mm.

E) Gene Ontology (GO) enrichment of genes among the second gene panel.

F-H) Heat maps showing individual scores of untreated cells and budding cells in enrichment of stress (**E**), senescence (**F**) and stemness (**G**) related gene sets. Student's *t* test.

I) Gene set enrichment analysis (GSEA) enrichment plots of genes in embryonic stem cells, signaling by Hedgehog, and signaling by Notch4 in budding RTP cells (compared with unirradiated cell).

J) Single-cell analysis of CD44 transcriptional expression in 5 stages; Kruskal-Wallis test.

K) Immunofluorescence staining of CD44v6 in pre-budding and budding RTP states of HCT116 cells (compared with unirradiated cell). Scale bar, 25mm. Integrated density (intDen) of stained CD44v6 is plotted; one-way ANOVA.

L-M) Immunohistochemistry (CD44v6) analysis of tumors for HCT116-xenograft and PDX in unirradiated and irradiation-induced RTP state. RTP tumors of HCT116-xenograft and PDX were harvested on day 11 of 10Gy and day 7 of 2 times 8Gy, respectively. Scale bar, 50 mm.

[N], unirradiated cells; [P], pre-budding RTP cells; [B], budding RTP cells; [S], budded progenies; [R], repopulated cell. *p < 0.05, **p < 0.01, ***p < 0.001; n.s., not significant.

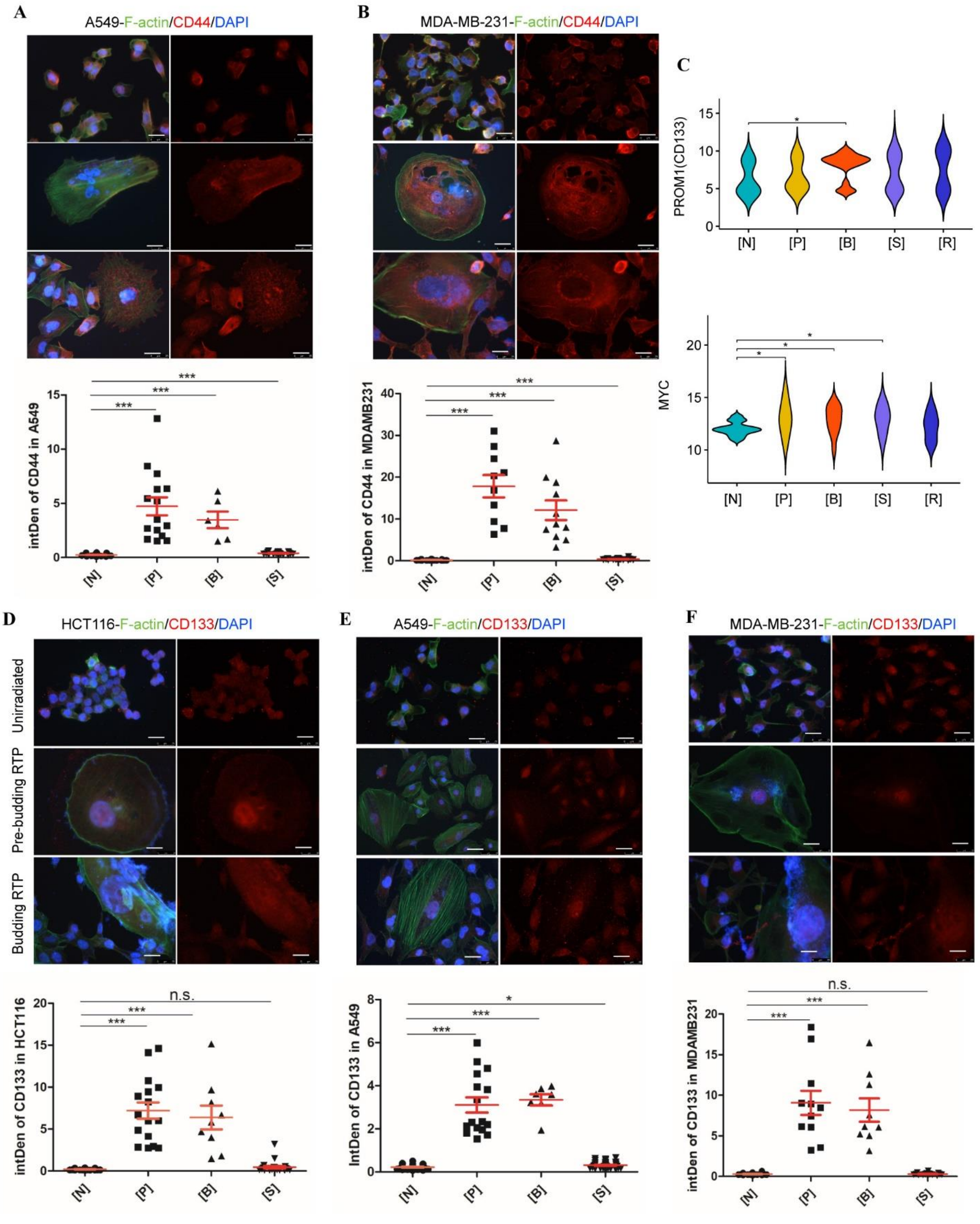
A
A549-F-actin/CD44/DAPI
intDen of CD44 in A549
***
***
***
0
5
10
15
[N]
[P]
[B]
[S]
B
MDA-MB-231-F-actin/CD44/DAPI
intDen of CD44 in MDAMB231
***
***
***
0
10
20
30
40
[N]
[P]
[B]
[S]
C
PROM1(CD133)
*
0
5
10
15
[N]
[P]
[B]
[S]
[R]
MYC
*
*
*
10
15
20
[N]
[P]
[B]
[S]
[R]
D
HCT116-F-actin/CD133/DAPI
Unirradiated
Pre-budding RTP
Budding RTP
intDen of CD133 in HCT116
n.s.
***
***
0
5
10
15
20
[N]
[P]
[B]
[S]
E
A549-F-actin/CD133/DAPI
IntDen of CD133 in A549
*
***
***
0
2
4
6
8
[N]
[P]
[B]
[S]
F
MDA-MB-231-F-actin/CD133/DAPI
intDen of CD133 in MDAMB231
n.s.
***
***
0
5
10
15
20
[N]
[P]
[B]
[S]

Figure S4. Expression profiling of traditional cancer stem cell markers

A-B) Immunofluorescence staining of CD44 in pre-budding and budding RTP states of A549 and MDA-MB-231 cells (compared with unirradiated cell). Scale bar, 25mm. Integrated density (intDen) of stained CD44 are plotted; one-way ANOVA.

C) Single-cell analysis of CD133 and MYC transcriptional expression in 5 cellular stages; Kruskal-Wallis test.

D-F) Immunofluorescence staining of CD133 in pre-budding and budding RTP states of HCT116, A549 and MDA-MB-231 cells (compared with unirradiated cell). Scale bar, 25mm. Integrated density of stained CD133 are plotted; one-way ANOVA.

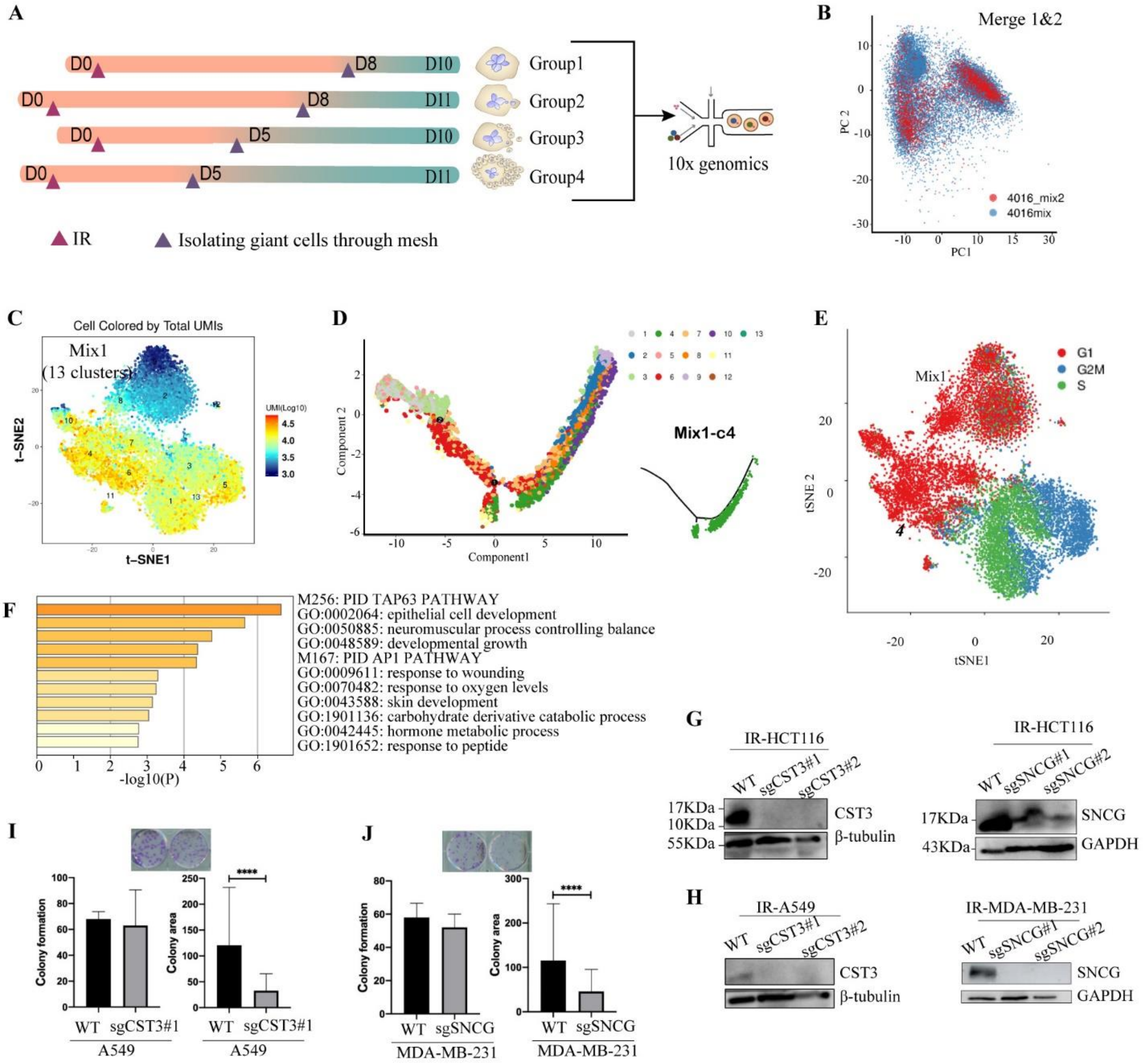

Figure S5. Verification by 10× scRNA-sequencing and by functional studies.

A) Supplementary diagram for scRNA-seq pipeline in **Figure 6A.** HCT116 RTP cells in different stages of budding were mixed up as first sample, consisting of 4 groups: giant cells isolated on D8 and cultivated till D10 (group1), giant cells isolated on D8 and cultivated till D11 (group2), giant cells isolated on D5 and cultivated till D10 (group3) and giant cells isolated on D5 and cultivated till D11 (group4).

B) t-SNE projections of merged mix1 (marked as blue) and mix2 (red).

C) t-SNE projections of single-cell RNA profiles in sample "Mix1". Cells were colored by total unique molecular identifiers (UMI).

D) Pseudo-time reconstruction of "Mix1" and cluster 4 located at the turning of "Mix1".

E) t-SNE projections of "Mix1" single cells colored by indicated cell cycle phases. Cluster 4 was located in G1 phase.

F) Gene Ontology (GO) terms of cluster 4 in "Mix1".

G-H) Western blot showing knockout of CST3 and SNCG in HCT116, as well as knockout of CST3 in A549 and SNCG in MDA-MB-231.

I-J) Colony formation capacity of untreated A549 cells and its CST3 knockout cells as well as untreated MDA-MB-231 cells and its SNCG knockout cells. Mean number of colonies were shown; Student's *t* test.

*p < 0.05, **p < 0.01, ***p < 0.001; n.s., not significant

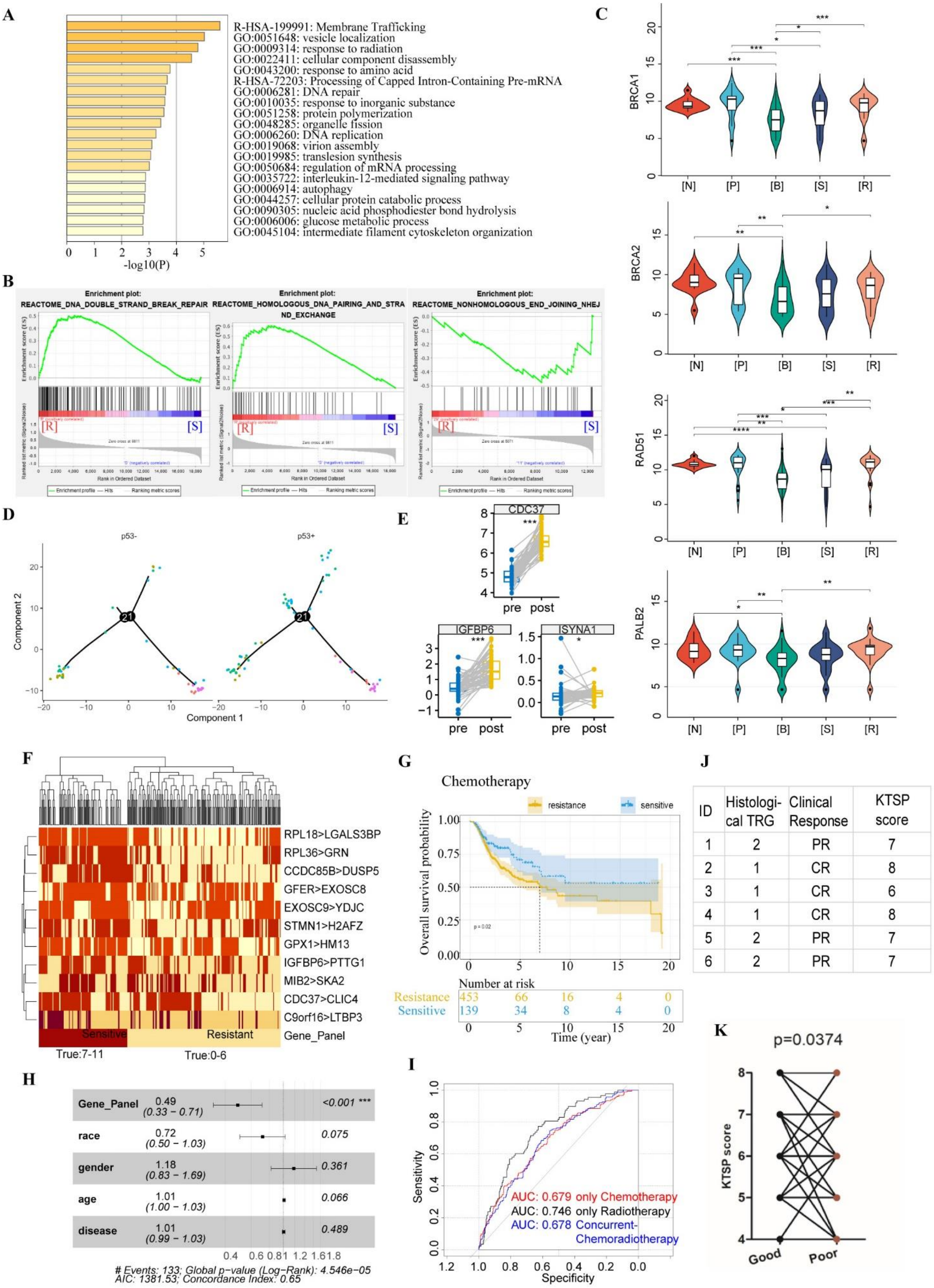
A
R-HSA-199991: Membrane Trafficking
GO:0051648: vesicle localization
GO:0009314: response to radiation
GO:0022411: cellular component disassembly
GO:0043200: response to amino acid
R-HSA-72203: Processing of Capped Intron-Containing Pre-mRNA
GO:0006281: DNA repair
GO:0010035: response to inorganic substance
GO:0051258: protein polymerization
GO:0048285: organelle fission
GO:0006260: DNA replication
GO:0019068: virion assembly
GO:0019985: translesion synthesis
GO:0050684: regulation of mRNA processing
GO:0035722: interleukin-12-mediated signaling pathway
GO:0006914: autophagy
GO:0044257: cellular protein catabolic process
GO:0090305: nucleic acid phosphodiester bond hydrolysis
GO:0006006: glucose metabolic process
GO:0045104: intermediate filament cytoskeleton organization
-log10(P)
B
Enrichment plot: REACTOME_DNA_DOUBLE_STRAND_BREAK_REPAIR
Enrichment plot: REACTOME_HOMOLOGOUS_DNA_PAIRING_AND_STRAND_EXCHANGE
Enrichment plot: REACTOME_NONHOMOLOGOUS_END_JOINING_NHEJ
[R]
[S]
Rank in Ordered Dataset
C
BRCA1
BRCA2
RAD51
PALB2
[N]
[P]
[B]
[S]
[R]
D
p53-
p53+
Component 1
Component 2
E
CDC37
IGFBP6
ISYNA1
pre
post
F
RPL18>LGALS3BP
RPL36>GRN
CCDC85B>DUSP5
GFER>EXOSC8
EXOSC9>YDJC
STMN1>H2AFZ
GPX1>HM13
IGFBP6>PTTG1
MIB2>SKA2
CDC37>CLIC4
C9orf16>LTBP3
Gene_Panel
Sensitive
Resistant
True:7-11
True:0-6
G
Chemotherapy
resistance
sensitive
Overall survival probability
p = 0.02
Number at risk
Resistance 453 66 16 4 0
Sensitive 139 34 8 4 0
Time (year)
J
ID | Histological TRG | Clinical Response | KTSP score
1 | 2 | PR | 7
2 | 1 | CR | 8
3 | 1 | CR | 6
4 | 1 | CR | 8
5 | 2 | PR | 7
6 | 2 | PR | 7
H
Gene_Panel 0.49 (0.33 – 0.71) <0.001 ***
race 0.72 (0.50 – 1.03) 0.075
gender 1.18 (0.83 – 1.69) 0.361
age 1.01 (1.00 – 1.03) 0.066
disease 1.01 (0.99 – 1.03) 0.489
Events: 133; Global p-value (Log-Rank): 4.546e−05
AIC: 1381.53; Concordance Index: 0.65
I
AUC: 0.679 only Chemotherapy
AUC: 0.746 only Radiotherapy
AUC: 0.678 Concurrent-Chemoradiotherapy
Sensitivity
Specificity
K
p=0.0374
KTSP score
Good
Poor

Figure S6.Reversibilityof RTP state and clinical value of the budding gene signature.

A) Pathway enrichment analysis of the amplified genes remained in repopulated [R] cells comparing with unirradiated [N] cells (located on 8q, 9q, 11 and 17q region).

B) GSEA showing positive enrichment for genes in "DNA double strand break repair" and "homologous DNA pairing and strand exchange", but negative enrichment for "non-homologous end joining" gene signatures in repopulated [R] cells comparing with newly budding [S] cells.

C) Transcriptional expression of homologous recombinant repair genes BRCA1, BRCA2, RAD51 and PALB2 in 5 cellular stages; Kruskal-Wallis test.

D) Trajectory analysis for HCT116 p53 (+/+) and p53 (-/-) samples.

E) Paired box diagrams showing the increase of [B]_type genes expression of 52 LARC patients following chemo-radiation (published data in GSE94104 and GSE190826).

F) Heat map showing the rule of top-scoring-pair (TSP) votes in predicting patient prognosis. For each pair of TSP with expression of gene A>B, the true score gets one point. When the cumulative true score is more than 7, prognosis of this patient is considered "sensitive"; otherwise, this patient is assigned to "resistant".

G) Overall Kaplan-Meier plot for cancer patients received chemotherapy from TCGA database, stratified to resistant and sensitive groups according to their individual KSTP scores grounding on budding gene panel (p=0.015, log rank test).

H) Forest plot illustrating that budding gene set acts as a risk factor in overall survival prediction for patients received radiotherapy only.

I) Logistics regression model showing the sensitivity and specificity of 11TSPs classifier in TCGA patients.

J) Relation between the clinic-pathological characteristics of LARC patients and their 11TSPs scores. TRG 1, single cells or small groups of cancer cells; TRG 2, residual tumors with evident tumor regression. CR, complete response; PR, partial response.

K) Line chart showing comparison of KTSP scores between 32 pairs of transcriptome. Thereinto, 24 pairs derived from locoregionally advanced nasopharyngeal cancer patients with

or without metastasis after radical radiotherapy; 8 pairs were from glioblastoma PDXs with acquired radio-resistance and untreated radio-sensitive status. Paired t test.

Supplemental materials and methods

Colony formation assay: Clonogenic assay was performed using the published instructions. [1] Briefly, cells were irradiated with different doses of X-rays and then seeded at different numbers (200 for 0Gy, 400 for 2Gy, 2500 for 4Gy, 5000 for 6Gy, 50,000 for 8Gy and 100,000 for 10Gy) in triplicate in 6-well plates. For colony formation of giant cells, 50,000 or 10,000 cells were seeded. When colonies were clearly visible after 12-14 days, cells were fixed and stained with 0.5% crystal violet. Colonies of more than 50 cells were enumerated and survival fractions were then calculated.

For the soft agar assay, tumor cells were suspended in a soft agar upper layer (0.35% agarose), which were overlaid on the lower layer (0.6% agarose) in 6-well plates. The colonies were then stained with 0.005% crystal violet and counted with Image J software. To evaluate the contribution of PACC in clonogenic formation after irradiation, clonogenic survival assay (at a density of 30,000 cells per well) and soft agar assay (5000 cells per well) were applied simultaneously for both giant and small cells separated at day 6.

Flow cytometry analysis: Dissociated cells were suspended in cell staining buffer to a concentration of 1×10^7 cells/ml. Cells were stained with 7-AAD (BioLegend, 420403), PE-conjugated antibodies for human CD133 (BD Biosciences, 566593) and APC-conjugated antibodies for human CD44 (BioLegend, 338805) on ice to incubate for 20 min. Following two

washes with PBS, cells were examined by flow cytometry (FACS Canto II, BD Biosciences) and the results analyzed with FlowJo software. The experiment was repeated twice to ensure consistence.

Karyotyping analysis: For metaphase preparation, when confluency of cultivated cells reached 80%, colcemid (0.2 μg/ml; Roche) was added to the culture medium for 3 hours in 37°C incubator. Afterwards, cells were centrifuged and incubated with 0.075mol/L KCl at 37°C for 20 min. Samples were then fixed three more times with fixation (3:1, methanol: glacial acetic acid) and every fixation lasted for 20 min. Cells were subsequently dropped onto an icy slide and air dried. Chromosome G banding and Karyotype analysis was performed using an LABB-M9 microscope.

EdU cell proliferation assay: EdU Cell Proliferation Assay (EdU-647, Sigma-Aldrich) was used per manufacturer's instructions. In short, cells were pulsed for 2 hours before harvesting by directly adding EdU solution to the cultured complete media. After cell fixation using 4% paraformaldehyde and permeabilization with 0.1% Triton X-100, cells were subjected to EdU click reaction. Combined Edu staining and IF was performed by subsequently blocked with 5% BSA in PBS and incubation of appropriate primary and secondary antibodies as described above. Images were acquired by fluorescence microscope (Leica).

Senescence detection: B-gal staining was performed using Senescence β-Galactosidase Staining Kit (CST, 9860) per manufacturer's protocol. Cells were fixed in a 4% paraformaldehyde and 0.2% glutaraldehyde solution for 15 min, and stained overnight at 37°C with an X-gal staining solution. For co-staining with other markers, a commercial SPiDER-β-

Gal kit (Dojindo) was used for fluorescent imaging according to the manufacturer's instruction. Briefly, after the incubation of primary antibody, SPiDER-β-Gal solution together with the secondary antibody was added to fixed cells. Images with bright field or fluorescence were collected by microscope (Leica) and counted with ImageJ software.

RNA sequencing data analysis:

For **SMART RNA-sequencing**, raw reads from Illumina sequencing were initially trimmed and filtered by fastp.[2] The clean paired-end reads were mapped to the reference genome *GRCh38* by HISAT2 (v 2.0), [3] and StringTie was used to assemble transcripts and estimate gene expression level.[4] FPKM value (Fragments Per Kilobase of transcript per Million mapped reads) was determined using RSEM[5] and Bowtie2[6]. After data estimation using DEBrowser[7] and data filtering excluding batch effect, differentially expressed genes (DEGs) was computed with DESeq2 [8] with a log2 Fold Change cutoff of 1 and an adjusted p-value cutoff of 0.01. Volcano plots, Venn plots and violin plots were performed using Enhanced Volcano (v1.7.16), VennDiagram (v1.6.20), vioplot (v0.3.5) and patchwork (v1.1.1). Functional enrichment analysis, including GO (Gene ontology) analysis and KEGG (Kyoto Encyclopedia of Genes and Genomes) analysis, was performed with ClusterProfiler. Fisher's exact test was applied to identify the significant pathway categories and FDR was used to correct the p-values. Gene Set Enrichment Analysis (GSEA) was performed using GSEA software (v4.0.3) (www.broadinstitute.org/gsea) using Hallmark Gene Sets from MSigDB (Broad Institute).[9] The Monocle2 package (v2.18.0) was used for pseudotime analysis indicating the evolvement from different stages.[10] Analysis of chromosome copy number

alteration was inferred using the R package inferCNV (v1.0.4).

For **10x single-cell RNA-sequencing**, raw sequencing data were filtrated and aligned to the reference genome *GRCh38* using CellRanger pipeline (10x Genomics, v3.1.0). For downstream processing, Seurat package (v.2.3) [11] (https://satijalab.org/seurat) was used for quality control, cellular filtration, data normalization, clustering and DEG analysis. Only cells that expressed at least 200 genes and had less than 10% of mitochondrial unique molecular identifiers (UMI) counts were kept for further processing. After removing low-quality data, the filtered matrix was performed log2 standardization. To reduce the high-dimensional transcriptome, principal component analysis (PCA) was performed on the highly varying genes. The top 20 principle components were then chosen for further tSNE (t-Distributed Stochastic Neighbor Embedding) clustering and visualization using the RunTSNE and TSNEPlot function of Seurat. To characterize these clusters, DEG between consecutive clusters were identified with Limma and thresholds for differential expression were fold-change≥ 1.5 and FDR≤0.05.[12] GO and KEGG pathway analysis were carried out using the ClusterProfiler R package. Cell cycle phases were computed based on gene expression profile of cell cycle using Cyclone function.[13] For single-cell pseudotime trajectories, Monocle 2 was performed with the normalized expression data. [10]

For quality control of bulk RNA-seq data, we firstly applied fastp filtering the adaptor sequence and removed the low quality reads. [2] Clean data were then mapped to the reference genome (GRCh38) by HISAT2 (v 2.0) with default parameters. [14] Gene expression levels of the transcripts were estimated by HTSeq (v 0.6.1). RPKM/FPKM (Reads/ Fragments Per Kilobase

Million Reads) was used to standardize the matrix data. DEseq2 was sequentially used to filter the DEG and significant DEG was defined as i), log2FC > 0.585 or < -0.585; ii), FDR < 0.05.[8] To evaluate the enrichment scores for each sample, single-sample GSEA (ssGSEA) was implemented using GSVA (v 1.30.0) package with ssGSEA method.[15] The molecular signatures used (including stress [16], senescence [17] and stemness [18] related gene set and EpiHR [19] signature) were listed in **Table.6**. The stem cell index [20], including CBC and RSC index was calculated using the R package ISCindex (https://github.com/gnvalbuena/ ISCindex).

Quantitative real-time polymerase chain reaction (qRT-PCR)

Tumor contents of FFPE tissue sections were evaluated and validated by a pathologist. Tumor RNA was then extracted from the FFPE sections using FFPE RNA Extraction Kit (AmoyDx), quantified and qualified using NanoDrop Spectrophotometer (Thermo Scientific) and reverse transcription into cDNA was performed with the PrimeScript™ RT Master Mix Kit (Takara). qPCR was performed with TB Green® Premix Ex Taq™ Kit (Takara) following the manufacturer's instructions. The primers used were listed in **Table 2.** Relative gene expression was analyzed using the comparative method ($2^{-\triangle\triangle CT}$) and results were obtained at three independent experiments.

Public datasets used: The treatment details and transcript profiles of residual tumor models used in Figure S2I were obtained from the publicly available GEO database GSE162285. [21] For each model, we examined their ssGSEA scores with transcript expression (residual *versus* untreated baseline) enriched in molecular profile or gene sets extracted from literature. [16a, 22] The rectal cancer transcriptomes pre- and post-neoadjuvant chemo-radiation used in Figure7G-

H were downloaded from GEO with accession numbers GSE94104 and GSE190826. Integration of array and RNA-seq datasets were performed using Rank-In (http://www.badd-cao.net/rank-in/index).

Datasets of oncotherapy-treated patients including transcript matrix and clinical annotations, were acquired from The Cancer Genome Atlas Program (TCGA) database with the R package TCGAbiolinks (v 2.13.3). Based on their courses of treatment, the TCGA cases covering 29 cancer types were divided into the following 3 groups: radiotherapy only (368 patients), chemotherapy (592 patients) and concurrent chemo-radiation (207 patients). Among patients received radiotherapy only, according to their clinical response status, they were classified as sensitive to radiation, including clinical complete response (CR), partial response (PR) and stable disease, or assigned to resistance to radiation with radiographic progressive or recurrent diseases.

The treatment details and transcriptome of nasopharyngeal cancer patients and glioblastoma PDXs in Figure S6K were obtained from dataset GSE103611[23] and GSE206225[24].

K-Top-scoring Pair classifier (*KTSP*): The *KTSP* algorithm provides a rank-based approach for classification, which has been developed successfully for predicting treatment response in several cancer types.[25] In this study, we firstly identified 48 DEG of budding RTP cells relative to untreated baseline grounding on the SMART-seq results. To simplify the 48-budding DEG in prognostic prediction, we enrolled 368 patients only received radiotherapy as training set to record the relative ordering of each pair of genes in the budding DEG. Based on clinical response of these patients, 11 top-scoring gene pairs were built for prognostic estimation. Each

pair of genes "votes" for poor prognosis and the final score is the sum of all votes among all 11 pairs. If the final score is less than 7, the patient is then assigned to sensitive group; otherwise, the patient is predicted to be resistant to treatment. Kaplan-Meier model and log rank test were then used to evaluate the effect of 11TSPs classifier on survival prediction in all three TCGA cohorts. A multivariate Cox proportional hazards regression model was constructed to evaluate whether the 11TSPs classifier was an independent prognostic factor. Logistic regression models using R package *glmnet* was performed to assess the efficiency of 11TSPs classifier in prognostic prediction.

References

[1] N. A. Franken, H. M. Rodermond, J. Stap, J. Haveman, C. van Bree, *Nat Protoc* **2006**, *1* (5), 2315, https://doi.org/10.1038/nprot.2006.339.

[2] S. Chen, Y. Zhou, Y. Chen, J. Gu, *Bioinformatics* **2018**, *34* (17), i884, https://doi.org/10.1093/bioinformatics/bty560.

[3] M. Pertea, D. Kim, G. M. Pertea, J. T. Leek, S. L. Salzberg, *Nat Protoc* **2016**, *11* (9), 1650, https://doi.org/10.1038/nprot.2016.095.

[4] S. Kovaka, A. V. Zimin, G. M. Pertea, R. Razaghi, S. L. Salzberg, M. Pertea, *Genome Biol* **2019**, *20* (1), 278, https://doi.org/10.1186/s13059-019-1910-1.

[5] B. Li, C. N. Dewey, *Bmc Bioinformatics* **2011**, *12*, 323, https://doi.org/10.1186/1471-2105-12-323.

[6] B. Langmead, S. L. Salzberg, *Nat Methods* **2012**, *9* (4), 357, https://doi.org/10.1038/nmeth.1923.

[7] A. Kucukural, O. Yukselen, D. M. Ozata, M. J. Moore, M. Garber, *BMC Genomics* **2019**, *20* (1), 6, https://doi.org/10.1186/s12864-018-5362-x.

[8] M. I. Love, W. Huber, S. Anders, *Genome Biol* **2014**, *15* (12), 550, https://doi.org/10.1186/s13059-014-0550-8.

[9] G. Yu, L. G. Wang, Y. Han, Q. Y. He, *OMICS* **2012**, *16* (5), 284, https://doi.org/10.1089/omi.2011.0118.

[10] X. Qiu, Q. Mao, Y. Tang, L. Wang, R. Chawla, H. A. Pliner, C. Trapnell, *Nat Methods* **2017**, *14* (10), 979, https://doi.org/10.1038/nmeth.4402.

[11] T. Stuart, A. Butler, P. Hoffman, C. Hafemeister, E. Papalexi, W. M. Mauck, 3rd, Y. Hao, M. Stoeckius, P. Smibert, R. Satija, *Cell* **2019**, *177* (7), 1888, https://doi.org/10.1016/j.cell.2019.05.031.

[12] D. J. McCarthy, Y. Chen, G. K. Smyth, *Nucleic Acids Res* **2012**, *40* (10), 4288, https://doi.org/10.1093/nar/gks042.
[13] A. Scialdone, K. N. Natarajan, L. R. Saraiva, V. Proserpio, S. A. Teichmann, O. Stegle, J. C. Marioni, F. Buettner, *Methods* **2015**, *85*, 54, https://doi.org/10.1016/j.ymeth.2015.06.021.
[14] D. Kim, B. Langmead, S. L. Salzberg, *Nat Methods* **2015**, *12* (4), 357, https://doi.org/10.1038/nmeth.3317.
[15] D. A. Barbie, P. Tamayo, J. S. Boehm, S. Y. Kim, S. E. Moody, I. F. Dunn, A. C. Schinzel, P. Sandy, E. Meylan, C. Scholl, S. Frohling, E. M. Chan, M. L. Sos, K. Michel, C. Mermel, S. J. Silver, B. A. Weir, J. H. Reiling, Q. Sheng, P. B. Gupta, R. C. Wadlow, H. Le, S. Hoersch, B. S. Wittner, S. Ramaswamy, D. M. Livingston, D. M. Sabatini, M. Meyerson, R. K. Thomas, E. S. Lander, J. P. Mesirov, D. E. Root, D. G. Gilliland, T. Jacks, W. C. Hahn, *Nature* **2009**, *462* (7269), 108, https://doi.org/10.1038/nature08460.
[16] a) C. Duy, M. Li, M. Teater, C. Meydan, F. E. Garrett-Bakelman, T. C. Lee, C. R. Chin, C. Durmaz, K. C. Kawabata, E. Dhimolea, C. S. Mitsiades, H. Doehner, R. J. D'Andrea, M. W. Becker, E. M. Paietta, C. E. Mason, M. Carroll, A. M. Melnick, *Cancer Discov* **2021**, *11* (6), 1542, https://doi.org/10.1158/2159-8290.CD-20-1375; b) T. Boroviak, R. Loos, P. Lombard, J. Okahara, R. Behr, E. Sasaki, J. Nichols, A. Smith, P. Bertone, *Dev Cell* **2015**, *35* (3), 366, https://doi.org/10.1016/j.devcel.2015.10.011.
[17] a) I. Ben-Porath, M. W. Thomson, V. J. Carey, R. Ge, G. W. Bell, A. Regev, R. A. Weinberg, *Nat Genet* **2008**, *40* (5), 499, https://doi.org/10.1038/ng.127; b) A. Pribluda, E. Elyada, Z. Wiener, H. Hamza, R. E. Goldstein, M. Biton, I. Burstain, Y. Morgenstern, G. Brachya, H. Billauer, S. Biton, I. Snir-Alkalay, D. Vucic, K. Schlereth, M. Mernberger, T. Stiewe, M. Oren, K. Alitalo, E. Pikarsky, Y. Ben-Neriah, *Cancer Cell* **2013**, *24* (2), 242, https://doi.org/10.1016/j.ccr.2013.06.005; c) A. L. Fridman, M. A. Tainsky, *Oncogene* **2008**, *27* (46), 5975, https://doi.org/10.1038/onc.2008.213.
[18] a) N. P. Palmer, P. R. Schmid, B. Berger, I. S. Kohane, *Genome Biol* **2012**, *13* (8), R71, https://doi.org/10.1186/gb-2012-13-8-r71; b) M. Ramalho-Santos, S. Yoon, Y. Matsuzaki, R. C. Mulligan, D. A. Melton, *Science* **2002**, *298* (5593), 597, https://doi.org/10.1126/science.1072530; c) A. Miranda, P. T. Hamilton, A. W. Zhang, S. Pattnaik, E. Becht, A. Mezheyeuski, J. Bruun, P. Micke, A. de Reynies, B. H. Nelson, *Proc Natl Acad Sci U S A* **2019**, *116* (18), 9020, https://doi.org/10.1073/pnas.1818210116.
[19] A. Canellas-Socias, C. Cortina, X. Hernando-Momblona, S. Palomo-Ponce, E. J. Mulholland, G. Turon, L. Mateo, S. Conti, O. Roman, M. Sevillano, F. Slebe, D. Stork, A. Caballe-Mestres, A. Berenguer-Llergo, A. Alvarez-Varela, N. Fenderico, L. Novellasdemunt, L. Jimenez-Gracia, T. Sipka, L. Bardia, P. Lorden, J. Colombelli, H. Heyn, X. Trepat, S. Tejpar, E. Sancho, D. V. F. Tauriello, S. Leedham, C. S. Attolini, E. Batlle, *Nature* **2022**, *611* (7936), 603, https://doi.org/10.1038/s41586-022-05402-9.
[20] E. G. Vasquez, N. Nasreddin, G. N. Valbuena, E. J. Mulholland, H. L. Belnoue-Davis, H. R. Eggington, R. O. Schenck, V. M. Wouters, P. Wirapati, K. Gilroy, T. R. M. Lannagan, D. J. Flanagan, A. K. Najumudeen, S. Omwenga, A. M. B. McCorry, A. Easton, V. H. Koelzer, J. E. East, D. Morton, L. Trusolino, T. Maughan, A. D. Campbell, M. B. Loughrey, P. D. Dunne, P. Tsantoulis, D. J. Huels, S. Tejpar, O. J. Sansom, S. J. Leedham, *Cell Stem Cell* **2022**, *29* (8),

1213, https://doi.org/10.1016/j.stem.2022.07.008.
[21] E. Dhimolea, R. de Matos Simoes, D. Kansara, A. Al'Khafaji, J. Bouyssou, X. Weng, S. Sharma, J. Raja, P. Awate, R. Shirasaki, H. Tang, B. J. Glassner, Z. Liu, D. Gao, J. Bryan, S. Bender, J. Roth, M. Scheffer, R. Jeselsohn, N. S. Gray, I. Georgakoudi, F. Vazquez, A. Tsherniak, Y. Chen, A. Welm, C. Duy, A. Melnick, B. Bartholdy, M. Brown, A. C. Culhane, C. S. Mitsiades, *Cancer Cell* **2021**, *39* (2), 240, https://doi.org/10.1016/j.ccell.2020.12.002.
[22] a) J. W. Schoggins, *Annu Rev Virol* **2019**, *6*, 567, https://doi.org/10.1146/annurev-virology-092818-015756; b) W. M. Schneider, M. D. Chevillotte, C. M. Rice, *Annu Rev Immunol* **2014**, *32*, 513, https://doi.org/10.1146/annurev-immunol-032713-120231; c) H. Cheon, E. C. Borden, G. R. Stark, *Semin Oncol* **2014**, *41* (2), 156, https://doi.org/10.1053/j.seminoncol.2014.02.002.
[23] X.-R. Tang, Y.-Q. Li, S.-B. Liang, W. Jiang, F. Liu, W.-X. Ge, L.-L. Tang, Y.-P. Mao, Q.-M. He, X.-J. Yang, Y. Zhang, X. Wen, J. Zhang, Y.-Q. Wang, P.-P. Zhang, Y. Sun, J.-P. Yun, J. Zeng, L. Li, L.-Z. Liu, N. Liu, J. Ma, *The Lancet Oncology* **2018**, *19* (3), 382, https://doi.org/10.1016/s1470-2045(18)30080-9.
[24] C. T. Stackhouse, J. C. Anderson, Z. Yue, T. Nguyen, N. J. Eustace, C. P. Langford, J. Wang, J. R. t. Rowland, C. Xing, F. M. Mikhail, X. Cui, H. Alrefai, R. E. Bash, K. J. Lee, E. S. Yang, A. B. Hjelmeland, C. R. Miller, J. Y. Chen, G. Y. Gillespie, C. D. Willey, *JCI Insight* **2022**, *7* (16), https://doi.org/10.1172/jci.insight.148717.
[25] a) N. D. Price, J. Trent, A. K. El-Naggar, D. Cogdell, E. Taylor, K. K. Hunt, R. E. Pollock, L. Hood, I. Shmulevich, W. Zhang, *Proc Natl Acad Sci U S A* **2007**, *104* (9), 3414, https://doi.org/10.1073/pnas.0611373104; b) R. R. Weichselbaum, H. Ishwaran, T. Yoon, D. S. Nuyten, S. W. Baker, N. Khodarev, A. W. Su, A. Y. Shaikh, P. Roach, B. Kreike, B. Roizman, J. Bergh, Y. Pawitan, M. J. van de Vijver, A. J. Minn, *Proc Natl Acad Sci U S A* **2008**, *105* (47), 18490, https://doi.org/10.1073/pnas.0809242105; c) M. Raponi, J. E. Lancet, H. Fan, L. Dossey, G. Lee, I. Gojo, E. J. Feldman, J. Gotlib, L. E. Morris, P. L. Greenberg, J. J. Wright, J. L. Harousseau, B. Lowenberg, R. M. Stone, P. De Porre, Y. Wang, J. E. Karp, *Blood* **2008**, *111* (5), 2589, https://doi.org/10.1182/blood-2007-09-112730.
